# The *Drosophila toothrin* Gene Related to the *d4* Family Genes: An Evolutionary View on Origin and Function

**DOI:** 10.3390/ijms252413394

**Published:** 2024-12-13

**Authors:** Elena E. Kuvaeva, Roman O. Cherezov, Dina A. Kulikova, Ilya B. Mertsalov

**Affiliations:** Koltzov Institute of Developmental Biology of Russian Academy of Sciences, 26 Vavilov Street, 119334 Moscow, Russia; lena_kuv@mail.ru (E.E.K.); ro-tcherezov@yandex.ru (R.O.C.); dinakulikova@mail.ru (D.A.K.)

**Keywords:** *toothrin (tth)*, *d4* family genes, *Dpf1-3*, DPF3a, DPF3a-like

## Abstract

*D. melanogaster* has two paralogs, *tth* and *dd4*, related to the evolutionarily conserved *d4* family genes. In mammals, the family consists of *Dpf1-3*, encoding transcription co-factors involved in the regulation of development and cell fate determination. The function of *tth* and *dd4* in *Drosophila* remains unclear. The typical domain structure of the proteins encoded by the *d4* family consists of an N-terminal 2/3 domain (Requiem_N), a central Kruppel-type zinc finger, and a C-terminal D4 domain of paired PHD zinc fingers (DPFs). In *Drosophila*, both paralogs lack the Kruppel-type ZF, and *tth* encodes a protein that contains only Requiem_N. In contrast, vertebrate *Dpf1-3* paralogs encode all the domains, but some paralogs have specific splice isoforms. For example, the DPF3a isoform lacks the D4 domain necessary for histone reading. The occurrence of proteins without the D4 domain in mammals and flies implies functional significance and analogous roles across animal taxa. In this study, we reconstructed the evolutionary events that led to the emergence of *Drosophila tth* by analyzing the divergence of *d4* paralogs across different evolutionary lineages. Our genomic and transcriptomic data analysis revealed duplications and gene copy loss events. Among insects, gene duplication was only observed in Diptera. In other lineages, we found the specialization of paralogs for producing isoforms and further specialization for coding proteins with specific domain organizations. We hypothesize that this pathway is a common mechanism for the emergence of paralogues lacking the D4 domain across different evolutionary lineages. We, thus, postulate that TTH may function as a splice isoform of the ancestral single-copy gene, possibly a DPF3a-like isoform characteristic of related insect species. Our analysis provides insights into the possible impact of paralogue divergence, emphasizing the functional significance of the 2/3 domain and the potential roles of isoforms lacking the D4 domain.

## 1. Introduction

The *d4* gene family comprises evolutionarily conserved genes encoding structurally related proteins with typical domain organization: they contain the N-terminal 2/3 domain (Requiem_N), the central Kruppel-type C2H2 zinc finger, and the C-terminal D4 domain of double PHD zinc fingers [1]. In vertebrates, this family comprises three genes: *neuro-d4*, *ubi-d4/Requiem*, and *cer-d4* [2,3,4,5,6], or *dpf1*, *dpf2*, and *dpf3* (*double PHD fingers 1-3*), respectively (according to GeneBank and NCBI Nomenclature). *Neuro-d4* and *cer-d4* are differentially expressed in the mouse nervous system during development, while the expression pattern of *ubi-d4/Requiem* is ubiquitous [5,6]. Although *cer-d4/dpf3* is considered cerebellum-specific, it is also expressed in the mouse retina, testes, and heart, as well as in the somites of mice, chickens, and zebrafish during development [6,7].

The mammalian DPF1-3 proteins, or BAF45b/c/d, respectively, are mutually exclusive subunits of ATP-dependent SWI/SNF-like BAF chromatin remodeling complex [8,9]. In the developing nervous system, BAF45b/c subunits have been shown to be associated with neuronal nBAF complex, which is characteristic of differentiated postmitotic neurons but not neuronal progenitor cells [8]. It has been demonstrated that the double PHD fingers (D4 domain) of DPF3 interact with acetylated or methylated histones H3 and H4 [7,10]. Similarly, the double PHD fingers of DPF2 can bind acetylated H3 and H4 [11], highlighting the role of the D4 domain as a histone reader. It has been reported that by recruiting SWI/SNF-like chromatin remodeling complexes to the specific histone marks, DPF3 regulates skeletal and cardiac muscle development and DPF2 controls myeloid differentiation [7,11]. It has also been reported that D4 proteins (DPF2 and DPF3) can function as adaptors between the SWI/SNF complex and NF-κB transcription factors, acting as transcriptional coactivators in the non-canonical and canonical NF-κB pathways, respectively [12,13]. For the human Requiem protein (DPF2), the region involved in interaction with the NF-κB RelB: p52 heterodimer and the SWI/SNF Brm-type chromatin remodeling complex has been mapped on the 2/3 domain (referred to as the NLS-containing N-terminal region) by in vitro assay [12], demonstrating the key role of the 2/3 domain in this dual interaction.

In addition to full-length proteins, mammalian *d4* family genes are capable of producing protein isoforms generated by alternative splicing. The *neuro-d4/Dpf1* can produce an isoform with an N-terminally truncated 2/3 domain, whereas *cer-d4/Dpf3* can generate a C-terminally truncated isoform with the loss of the D4 domain, an isoform DPF3a/XZ, which contains a specific structure comprising part of the first PHD finger of the D4 domain (1/2PHD1) with the adjacent alternative C-terminus [2,5,6,7]. The DPF3a isoform plays an important role in muscle development and in the onset of various pathologies. It has been reported that the DPF3a can regulate the transcription of genes involved in cardiac hypertrophy [14], myogenic differentiation [15], and kidney cancer cell migration [16] through interactions with transcription factors or chromatin regulators via its specific C-terminus.

In *Drosophila melanogaster*, we have previously identified two paralogs of the *d4* family. First, the *drosophila d4* (*dd4*, *CG2682*) gene, which encodes a protein with two conserved domains specific to the D4 family (the N-terminal 2/3 domain and the C-terminal D4 domain), has been specified as the sole member of the D4 family in *D. melanogaster* [17]. The second gene, designated “*toothrin*” (*tth*, *CG12175*) by virtue of encoding a protein that lacks a D4 domain but possesses a 2/3 domain, has been classified as a *d4*-related gene [18]. The dD4 (DD4) and TTH proteins of *D. melanogaster* have been shown to be associated with the BAP complex, which is homologous to the mammalian BAF chromatin remodeling complex [19]. The exact function of the *D. melanogaster d4* family genes remains elusive. Nevertheless, the expression pattern of *dd4* may suggest its potential involvement in the development of the nervous system and gonads [17]. A role for *tth* in the hormonal regulation of *Drosophila* development has been proposed [18].

The presence of proteins lacking the D4 domain in mammals and flies suggests functional importance and potential similar functions across different animal groups. We aimed to investigate the sequence of evolutionary events shaping the observed *d4* paralog divergence across evolutionary lineages to comprehend the evolutionary trend that resulted in the emergence of *Drosophila tth*. By employing ortholog databases and genomic/transcriptomic data analysis from NCBI, we reconstructed the evolutionary history of the *d4* gene family, identifying duplication events, structural divergence trends, and isoform formation patterns across vertebrates and invertebrates. We found that among insects, the only occurrence of duplication was observed in the Diptera. In different evolutionary lineages, we found the specialization of paralogs for the production of isoforms and further specialization for coding proteins with specific domain organizations. We proposed that *Drosophila tth* may assume the function of a splice isoform of the ancestral single-copy gene, potentially a DPF3a-like isoform characteristic closely related to the Diptera insect orders. Our analysis revealed the potential neofunctionalization of the 2/3 domains in Diptera paralogs, which may be related to their specialization in interacting with distinct factors, presumably the homologs of NF-κB transcription factors.

## 2. Results

### 2.1. D4 Family Genes in Diptera

**Figure 1:**
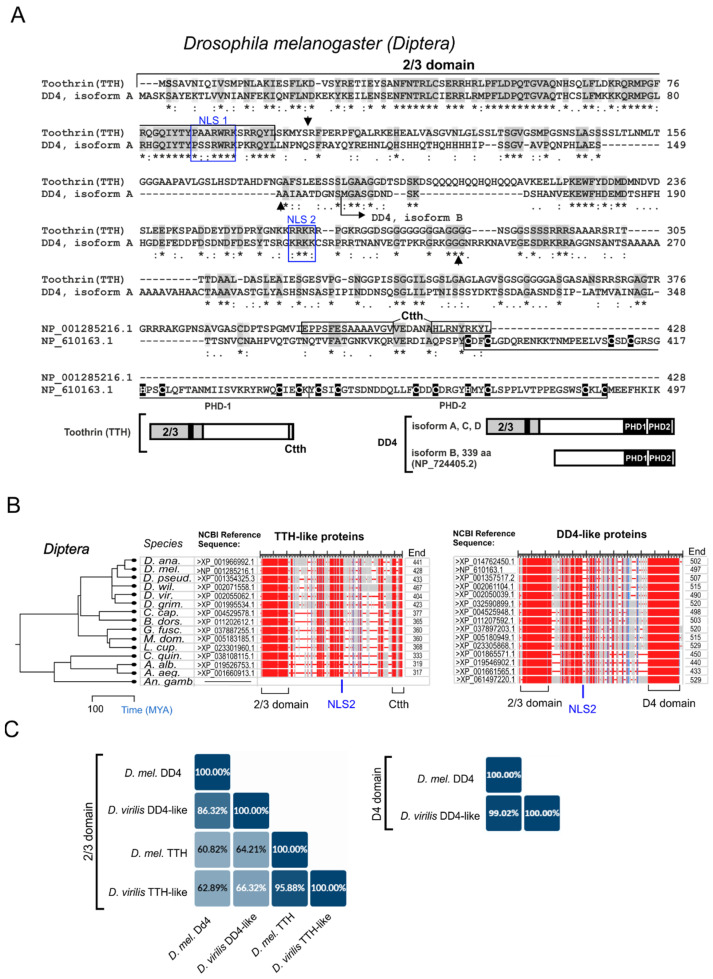
D4-related proteins in Diptera. (**A**) The Clustal Omega alignment of the amino acid sequences of *D. melanogaster* TTH (NP_001285216.1) and DD4 (NP_610163.1) proteins. (*)—conserved residue; (:)—scoring > 0.5; (.)—scoring ≤ 0.5. Brackets combine conserved domains. Arrowheads above and below sequences indicate the positions of introns in TTH and DD4, respectively. The C-terminal sequences in the TTH that are conserved in the Diptera are highlighted with black frames. Designations: 2/3 domain—N-terminal conserved domain characteristic for D4 family proteins; PHD1, PHD2—tandemly arranged pair of PHD zinc fingers forming the D4 domain, zinc-binding amino acids highlighted in black; NLS—putative nuclear localization signals are highlighted with blue frames. Below are the schematic images of the domain organization of TTH (NP_001285216.1) and DD4 (isoform A (NP_610163.1) and isoform B (NP_724405.2)). The 2/3 domain carrying NLS (black bar) is filled with gray, and the PHD fingers (PHD1, PHD2) are filled with black. (**B**) The NCBI Multiple Sequence Alignment Viewer’s graphical display of the TTH-like and DD4-like proteins sequence alignment. The scale long marks indicate every 50 positions along the alignment. The red regions indicate conserved sequences. The variable regions are highlighted in gray. The brackets indicate the location of the 2/3 and D4 domains. On the left side is a phylogram that demonstrates the relationships and estimated divergence times of the selected species. The species are as follows: *D. ana*—*D. ananassae*; *D. mel.*—*D. melanogaster*; *D. pseud.*—*D. pseudoobscura*; *D. wil.*—*D. willistoni*; *D. vir.*—*D. virilis*; *D. grim.*—*D. grimshawi*; *C. cap.*—*Ceratitis capitata*; *B. dors.*—*Bactrocera dorsalis*; *G. fusc.*—*Glossina fuscipes*; *M. dom.*—*Musca domestica*; *L. cup.*—*Lucilia cuprina*; *C. quin.*—*Culex quinquefasciatus*; *A. alb.*—*Aedes albopictus*; *A. aeg.*—*Aedes aegypti*; *An. gamb.*—*Anopheles gambiae*. (**C**) The percent identity matrix of the aligned 2/3 domains (left panel) and D4 domain (right panel) of the TTH and DD4 proteins of *D. melanogaster* (*D. mel.*) and *D. virilis* generated by the Clustal Omega program from the UniProt resources [20]. The colors from dark blue to light blue indicate the higher and lower similarity, respectively.

The characteristics of *D. melanogaster tth* and *dd4* retrieved from the NCBI gene models revealed that *tth* contains two exons, and produces a single protein of 428 a.a. (Figure S1). The first exon encodes a conserved 2/3 domain characteristic of the N-terminal region of the protein products of the *d4* family genes (Figure 1). The second exon encodes an amino acid sequence with a disordered structure containing short stretches of polar, basic, or acidic residues.

In contrast to *tth*, its paralog *dd4* has alternative transcription and splicing and generates four isoforms: isoforms A, C, and D, which encode a full-length protein (495-497 a.a.) bearing 2/3 and D4 domains, and isoform B, which encodes an N-terminally truncated protein (339 a.a.) without domain 2/3 (Figure 1A and Figure S1B). The protein products of *tth* and *dd4* lack C2H2 Kruppel-type zinc fingers (ZFs) (Figure 1A).

Therefore, the *tth* and *dd4* orthologs are distinguished by a specific combination of domains present in their protein products.

First, we reconstructed the evolutionary history of the *tth* and *dd4* genes in insects. For this, we performed an ortholog search using ORTHODB v11 [21]. To confirm orthologies, we employed BLASTP against the NCBI database where the amino acid sequences of TTH and DD4 (the 2/3 and D4 domains) were used as queries. During the search, we found that the sequence of the double PHD zinc fingers of the D4 domain has matches with other proteins containing double PHDs (orthologs of the PHF10 protein and the histone acetyltransferases MOZ and MORF). To avoid wrong matches with other double PHD-containing proteins, the search parameters was restricted to 50% identity at 100% coverage of the D4 domain query sequence. Our genomic survey clearly revealed that both *dd4* and *tth* paralogs are exclusive to the order Diptera, except for *Anopheles* species, which have been found to contain single-copy orthologs of *dd4* (Figure 1B, Table S1). We have identified 85 *dd4* orthologs in 85 Diptera species and 70 *tth* orthologs in 70 Diptera species (33 species are listed in Table S1). Single-copy *d4* family genes have been identified in the genomes of other insect species (Table S1). In contrast to the Dipteran *tth* and *dd4*, these genes encode all the signature domains of the D4 family proteins, including the Kruppel-type ZF. Thus, we concluded that the *tth* and *dd4* paralogs are unique to the Diptera and may have arisen as a result of the duplication of a single-copy *d4* gene of the Diptera ancestor.

To gain insight into the potential mechanism of gene duplication in Diptera, we conducted an analysis of gene structure for the *tth*-like and *dd4*-like orthologs of 33 Diptera species (Table S1). The analysis revealed the intronless structure of the coding regions in mosquitoes. In the *tth*-like and *dd4*-like orthologs of Brahysera (Muscomorpha) species, a consistent number of introns were found to be splitting coding exons. Specifically, one intron splits coding exons in the *tth*-like genes, while two split coding exons in the *dd4*-like genes. The positions of the introns are conserved for each paralog and do not overlap between them (Figure 1A), indicating that the introns originated independently after duplication. These findings suggest that the duplication of the *d4* family genes in the Diptera order is most likely the result of retroposition events.

To determine whether *Anopheles* inherited a single *dd4*-like gene from an ancestor or lost a previously duplicated copy during evolution, we conducted an evolutionary analysis of *dd4* and *tth* protein-coding sequences using the Maximum Likelihood method to estimate the paralogue distribution on the phylogenetic tree branches in 16 species of Diptera. The resulting tree demonstrates that the paralogs evolved similarly and independently in separate branches of Brachycera (Muscomorpha) and Nematocera (including mosquito) (Figure S2A). This indicates that the common ancestor of these two suborders possessed both paralogs. Therefore, it is highly likely that the absence of the *tth* paralog in *Anopheles* species is the consequence of gene loss.

To gain insight into the potential scenarios that may have resulted in the loss of the *tth*-like gene in *Anopheles*, we analyzed the arrangement of *tth* anchor genes provided by the NCBI genome browser in four mosquito species: *Aedes aegypti*, *Aedes albopictus*, *Culex quinquefasciatus*, and *Culex pipens pallens*. We used the DiGAlign software (version 2.0) to analyze the synteny between 200 kb genomic fragments containing *tth*-like loci. No or little synteny was found between the *Aedes* and *Culex tth* loci. This suggests high instability in regions adjacent to *tth*, which may be related to DNA transpositions. Next, BLASTP was used to search for the orthologs of the corresponding anchor genes in the genomes of *Anopheles gambiae* and *Anopheles albimanus*, which are separated by approximately 100 million years of evolutionary divergence [22]. All the orthologs of the *Aedes* and *Culex tth* anchor genes were found on the X chromosomes of these *Anopheles* species. The analysis of the arrangement of the corresponding orthologs on the chromosomes has led us to two possible scenarios for *tth*-like gene loss in *Anopheles*. The first scenario suggests that this gene may have been lost as a result of an inversion or deletion. This proposal is further supported by the positioning of the orthologs of the *Aedes tth* anchor genes in *Anopheles*, which in *Aedes* are situated upstream and downstream of the *tth* gene, while in *Anopheles*, they are in close proximity to each other (Figure S2B). Another potential scenario suggests that the *tth* locus in *Anopheles* may have been disrupted by multiple transposon insertions. The hypothesis is based on the findings that the orthologs of the *tth* anchor genes characteristic of *Culex* are located on the X chromosome of *Anopheles* at considerable distances from each other. Specifically, in *Anopheles albimanus*, the distance is approximately 3 Mb, while in *Anopheles gambiae*, it is approximately 11.5 Mb (Figure S2B).

The multiple sequence alignment of the deduced full-length amino acid sequences of DD4 and TTH paralogs from various Diptera species indicates high sequence conservation in the 2/3 and D4 domains. For instance, 15 species are shown in Figure 1B and Figure S1. The sequence similarity between 2/3 domains of TTH-like and 2/3 domains of DD4-like proteins in these species shows 64.95–95.88% identity and 63.54–97.89% identity, respectively. The similarity between the D4 domains of DD4-like proteins shows 87.25–99.02% identity. Two conserved putative nuclear localization signals, NLS1 and NLS2, were found in both paralogs (Figure 1A,B, Figures S1 and S3). The regions not associated with the domains are less conserved. However, both paralogs have short conserved motifs that are localized between domain 2/3 and the central region containing the putative nuclear localization signal 2 (NLS2) (Figure 1B and Figure S1), suggesting that the TTH and DD4 paralogs are related in their N-terminal halves (Figure 1A). In the C-termini of TTH-like proteins (amino acids 399-428 in *D. melanogaster*), there are conserved motifs 14 and 9 a.a. that are specific to these orthologs and show no similarity to any other amino acid sequence in the available databases (Figure 1 and Figure S1). We have designated it as a TTH-specific C-tail, or Ctth. Then, we noticed that the interspecies similarity between the 2/3 domains of the DD4 and TTH paralogs is higher than the similarity between 2/3 domains of the paralogs within the same species (Figure S4). For example, within the same species, the similarity between the 2/3 domains of two paralogs reaches 60.82% and 66.32% identity in *D. melanogaster* and *D. virilis*, respectively, whereas the interspecies similarity between 2/3 domains of the paralogs in *D. melanogaster* and *D. virilis* reaches 95.88% identity for TTH and 86.32% identity for DD4 (Figure 1C). This may suggest that the divergence between the 2/3 domains of the TTH and DD4 paralogs is functionally relevant according to the “ortholog conjecture”, which states that orthologous genes are functionally more similar than paralogous genes [23,24].

### 2.2. d4 Genes in Other Insects

**Figure 2:**
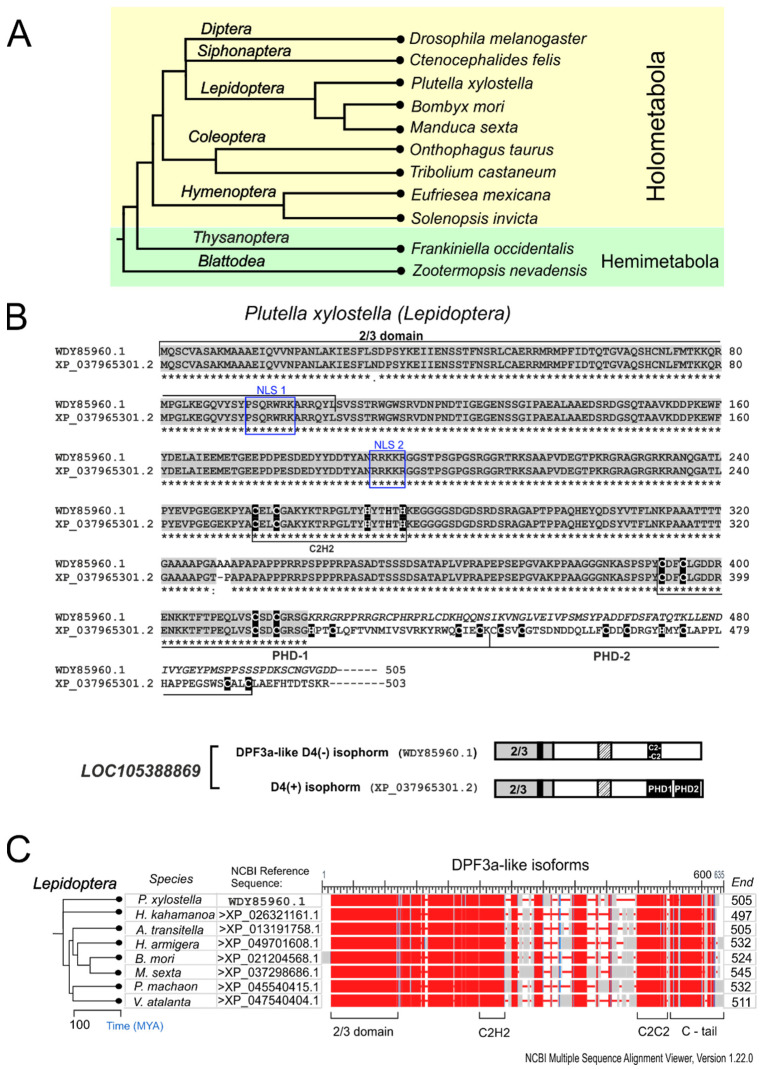
Organization of two protein isoforms produced by the *d4* family gene *LOC105388869* of *P. xylostella*. (**A**) A phylogram shows the relationships among insect species. (**B**) Alignment of the amino acid sequence of two protein isoforms generated by *d4*-related gene in *P. xylostella.* The identical regions are highlighted in gray. The mismatches reflect minimal differences between the predicted isoform XP_037965301.2 from the NCBI protein database and isoform WDY85960.1 translated from ORF of the alternative transcript OQ559524 confirmed in this work. (*)—conserved residue; (:)—scoring > 0.5; (.)—scoring ≤ 0.5. Conservative 2/3 domain, PHD1, PHD2, and C2H2 zinc fingers are bounded above and below the sequences by brackets. C-tail alternative to the D4 domain highlighted in italics. Designations: 2/3—2/3 domain; PHD1, PHD2—tandemly arranged pair of PHD zinc fingers forming the D4 domain, zinc-binding amino acid residues are highlighted in black; putative nuclear localization signals (NLS1 and NLS2) are framed in blue; C2H2—Kruppel-type zinc finger. The domain organization of two isoforms is shown below. The 2/3 domain carrying NLS (black bar) is filled with gray, the Kruppel-type zinc finger is shaded, and the PHD fingers and a C2C2 motif of PHD1 are filled with black. (**C**) The NCBI Multiple Sequence Alignment Viewer’s graphical display of the DPF3a-like isoform alignment of different Lepidoptera species. The scale long marks indicate every 50 positions along the alignment. The red regions indicate conservative sequences. The variable regions are highlighted in gray. The brackets indicate the location of the 2/3, C2H2—Kruppel-type zinc finger, and D4 domains. Species names: *P. xylostella* (*Plutella xylostella*), *H. kahamanoa* (*Hyposmocoma kahamanoa*), *A. transitella* (*Amyelois transitella*), *H. armigera* (*Helicoverpa armigera*), *B. mori* (*Bombyx mori*), *M. sexta* (*Manduca sexta*), *P. machaon* (*Papilio machaon*), and *V. atalanta* (*Vanessa atalanta*). A phylogram shows relationships and estimated divergence times among the selected species in the Lepidoptera order.

As we previously noted, only the Diptera species have two *d4*-related paralogs, while other insects possess a single-copy *d4*-related gene encoding a protein with all the D4 family-specific domains, including the Kruppel-type ZF. We then analyzed the protein products of the *d4* family genes in the Siphonaptera and Lepidoptera species available in the NCBI RefSeq database, as they are the closest related to Diptera orders (Figure 2A). The available genomic data for Siphonaptera species is limited to one species: the cat flea, *Ctenocephalides felis*. Two splice isoforms were identified in the gene model of this species: a full-length isoform and an isoform lacking a Kruppel-type ZF. In Lepidoptera species, however, in addition to the full-length proteins, we also found predicted isoforms that could be the result of alternative splicing that causes the loss of the D4 domain. These isoforms contain a 2/3 domain and a Kruppel-type ZF, but instead of a D4 domain, they have a C2C2 motif of PHD1 zinc finger (1/2 PHD1) with an adjacent alternative stretch of amino acids. The similarly organized isoforms, named XZ or DPF3a, have been previously described in the protein products of the vertebrate *Dpf3* (*Cer-d4*) gene [6,7]. We found such DPF3a-like isoforms among the predicted proteins of numerous Lepidoptera species whose genomes are available in NCBI (Table S1). According to the NCBI gene model for the *Bombyx mori d4* gene, the splicing pattern of DPF3a-like isoforms involves the elongation of the C2C2 motif coding sequence by the downstream extension of exon 9 into the adjacent intron followed by alternative transcription termination (EE/ATT) (Figure S5).

To verify the predicted isoform, we performed TBLASTN of the NCBI Transcriptome Shotgun Assembly Sequence Database (TSA) using the C-terminal protein sequence of *B. mori* DPF3a-like isoform as a query. We found several transcribed RNA fragments containing part of the coding sequence (e.g., 622 nt RNA fragment from *Bombyx mori* (acc# CK542553) and 1561 nt RNA fragment from *P. xylostella* (acc# GFRV01000142). The diamondback moth *Plutella xylostella* was chosen for transcript validation (Figure 2A). According to the *LOC105388869* NCBI gene model, the *P. xylostella d4* family gene consists of 12 exons encoding the full-length protein, of which exons 9–12 encode the D4 domain but no transcripts encoding DPF3a-like isoform have been annotated for this species. Using RT-PCR, we amplified a cDNA fragment containing full-length ORF (GenBank submission #OQ559524), encoding a *P. xylostella* DPF3a-like isoform (Figure 2B).

The multiple sequence alignment of the deduced amino acid sequences of DPF3a-like isoforms from eight Lepidoptera species revealed high conservation of the 2/3 domain, the Kruppel-type ZF, the region in between, and the C2C2 motif with the adjacent C-terminus, while the remaining region between the Kruppel-type ZF and the C2C2 motif contains a variable sequence (Figure 2C).

Next, we analyzed the protein products of the *d4* family genes of other insects from the Holometabola and Hemimetabola clades, whose genomes are available at NCBI (33 selected species are shown in Table S1). In contrast to Lepidoptera, we found a limited number of gene models with predicted isoforms that lack the D4 domain. We found that *d4* gene models from beetles/Coleoptera (*O. taurus* and *T. Castaneum*), ants and bees/Hymenoptera (*S. invicta*, *A. echinatior*, and *E. mexicana*), and Hemimetabola termite/Blattodea (*Z. nevadensis*) predict multiple splice isoforms encoding full-length proteins, proteins lacking Kruppel-type ZF, and DPF3a-like protein isoforms (Figure 2A). However, we found that only in *O. taurus*, *T. castaneum* (Coleoptera), and *E. mexicana* (Hymenoptera) the predicted DPF3a-like isoforms could be indirectly confirmed by the transcriptome data of the related species available in NCBI TSA (Figure S6).

**Figure 3:**
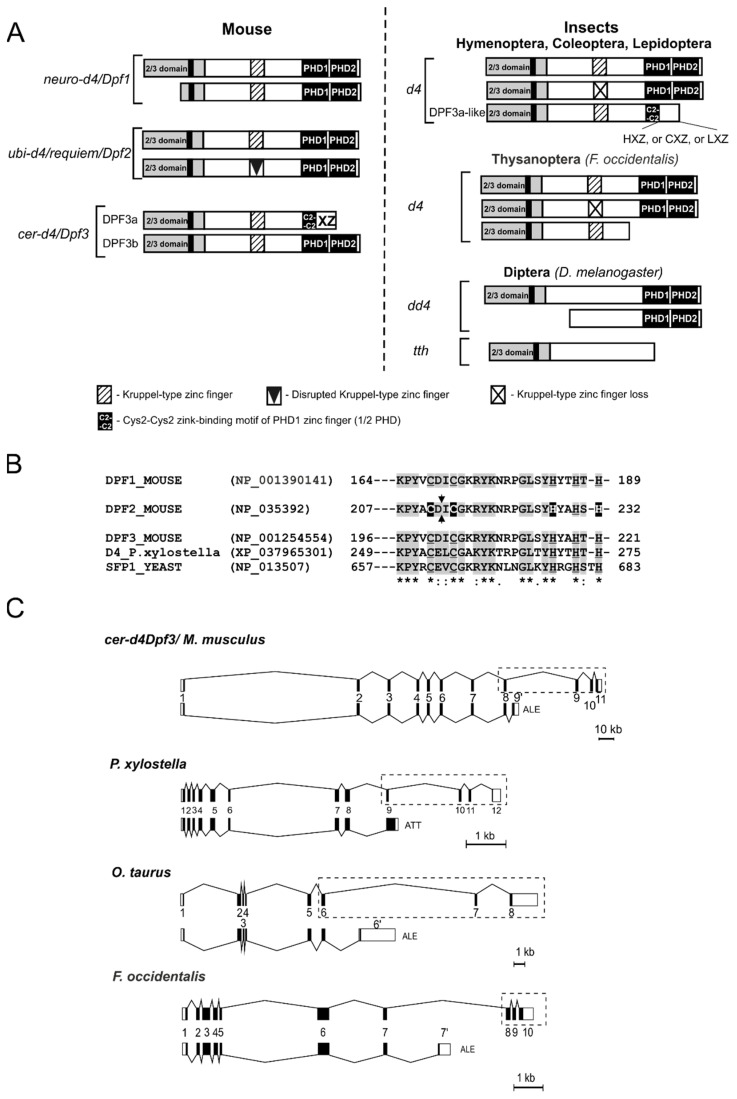
The D4 family proteins of mammals and insects. (**A**) The domain organization of D4 proteins produced by mouse *neuro-d4/Dpf1*, *ubi-d4/Requiem/Dpf2*, and *Cer-d4/Dpf3*, and insects *d4*-like genes. The 2/3 domain is filled with gray, and the black bar indicates NLS. The PHD1 and PHD2 fingers of the D4 domain are shown as black boxes. XZ, HXZ, CXZ, and LXZ—an alternative to D4 domain C-tails conserved in vertebrates (XZ) and Hymenoptera, Coleoptera, and Lepipotera insect orders, respectively. (**B**) The alignment of the amino acid sequences of C2H2 Kruppel-type zinc fingers of mouse and *P. xylostella* (Lepidoptera) D4 proteins and Sfp1 protein of yeast. (*)—conserved residue, (:)—scoring > 0.5, (.)—scoring ≤ 0.5. Two arrows indicate the position of 14 a.a. insertion (NSFKQKHTSKAPQR) specific for isoform in Ubi-d4/Requiem/DPF2. Positions of zinc-binding amino acids in black are indicated according to crystal structure of domain in DPF2 [25]. (**C**) The splicing patterns of the *d4* genes leading to the loss of the D4 domain, showing the positions of exons (vertical lines) and scale bars. The exons coding for the D4 domain are in dashed boxes. ALE—alternative last exon. ATT—alternative transcription termination.

The analysis of the NCBI RefSeq models of bee *E. mexicana* and beetle *T. castaneum d4* genes reveals that like in butterflies, their DPF3a-like isoforms are generated by an EE/ATT-type splicing characterized by the extension of the exon encoding the C2C2 motif of PHD1. However, the NCBI *d4* gene model for the beetle *O. taurus* reveals that the DPF3a-like isoforms are generated by an alternative last exon (ALE) inclusion splicing, which is similar to the type of splicing that generates the DPF3a isoforms in vertebrates (Figure 3 and Figure S5).

Thus, we can conclude that in different insects, the DPF3a-like isoform can result from two types of splicing: (1) exon extension /ATT and (2) ALE-type (Figure 3 and Figure S5).

In the *d4* gene models of the Hemimethabola clade, we found no TSA-confirmed DPF3a-like isoforms. However, the NCBI model of the *d4*-like gene in the Hemimethabola species *Frankliniella occidentalis* (Thysanoptera) shows that ALE-type splicing results in an isoform other than DPF3a-like. This isoform lacks all the exons encoding the D4 domain (Figure 3 and Figure S5).

The multiple sequence alignment of *D. melanogaster* TTH and DD4 proteins, the DPF3a-like isoforms from three species of the insect clade Holomethabola (*P. xylostella*, *O. taurus*, and *E. mexicana*), and isoform lacking the D4 domain from Hemimethabola *F. occidentalis* shows that additionally to the conserved 2/3 domain, there are short stretches of conserved a.a. between the 2/3 domain and a central region of basic residues containing the conserved NLS2 (Figure S7). This may indicate a high level of functional constraint of these regions in the D4 proteins of *Drosophila* and other insects. Both the Kruppel-type ZF and the C2C2 motif are conserved among the DPF3a-like isoforms. However, their C-termini differ significantly, and the sequence similarity may be found only among species of the same order (Figures S6 and S7). Moreover, within Coleoptera order in species belonging to the families Scarabaeidae (*O. taurus* and other) and Tenebrionidae (*T. castaneum* and other), the C-termini of the DPF3a-like isoforms are conserved for each taxonomic group (Figure S6). Since the C-termini of vertebrate Cer-d4/Dpf3 splice isoforms DPF3a were designated as XZ (according to [6]), we have designated them as LXZ, HXZ, and CXZ for Lepidoptera, Hymenoptera, and Coleoptera DPF3a-like isoforms, respectively, in insects (Figure 3 and Figure S6).

In summary, our analysis of the *d4* family genes in insect genomes from the NCBI RefSeq database reveals that only Diptera has two paralogs, *tth* and *dd4*, encoding proteins with domain composition specific to each paralogue, while other insects have a single copy of the *d4* family gene, capable of producing differently organized isoforms: a full-length protein and a truncated isoform lacking the D4 domain.

### 2.3. Evolutionary Analysis of the d4 Family Genes

Once we found that the *tth* orthologs in insects are unique to Diptera, to address whether there are genes in species of other taxonomic groups that, like *tth*, do not encode the D4 domain, we performed a genome-wide search for *d4* family orthologs in the NCBI database and examined the evolution of the *d4* family genes (121 species were selected to characterize the evolution of the *d4* gene family, Table S1). A review of the OrthoDB and NCBI Orthologs databases revealed that *d4* family genes are exclusive to metazoans. A TBLASTN search of the *d4* homologs in protozoan genomes revealed that single-celled organisms, including *Monosiga brevicollis* (a member of the Choanoflagellata, which is considered the evolutionary ancestor of sponges) and the single-/multi-celled social amoeba/mycetozoa *Dictyostelium purpureum*, possess genes encoding proteins that are not homologous with D4 proteins but do contain double PHD fingers (Table S1). These may be protozoan genes that are ancestral to the metazoan genes encoding double PHD-containing proteins, DPFs (D4/DPF1-3 family proteins, orthologs of PHF10 protein and histone acetyltransferases MOZ and MORF), as previously suggested for the amoeba Naegleria lovaniensis [26]. We found that non-bilaterians (placozoa, sponges, and coelenterates) and Protostomes (mollusks and worms) have a single *d4* family gene copy (Table 1).

In deuterostomes, a single-copy *d4* family gene is present in Echinodermata (*A. rubens*), Hemichordata (*S. kowalevskii*), Acrania in fillum Chordata (*B. floridae*), and Tunicata (*C. intestinalis*) genomes (Table 1). All of these single-copy *d4* family genes encode three signature domains of D4 proteins: a 2/3 domain, a Kruppel-type ZF, and a D4 domain.

The first duplications of the *d4* genes began to occur during the evolution of Arthropods (protostomes). For example, some crustacean species (*Daphnia magna*) have one gene of the *d4* family, while others (*Eurytemora affinis*, *Hyalella azteca*, *Penaeus japonicus*, *Homarus americanus*, and *Portunus trituberculatus*) have two. *Limulus polyphemus*, a horseshoe crab that represents an early evolutionary branch of the Chelicerae (Merostomata), has three paralogs. In the other Chelicerata, the arachnids, some species have a single *d4* gene (*Ixodes scapularis*), two (*Centruroides sculpturatus*), three (*Panonychus citri*), and some even four (*Tetranychus urticae*) (Table 1 and Table S1). In insects, the duplication of the *d4* family gene occurred only in Diptera.

**Figure 4:**
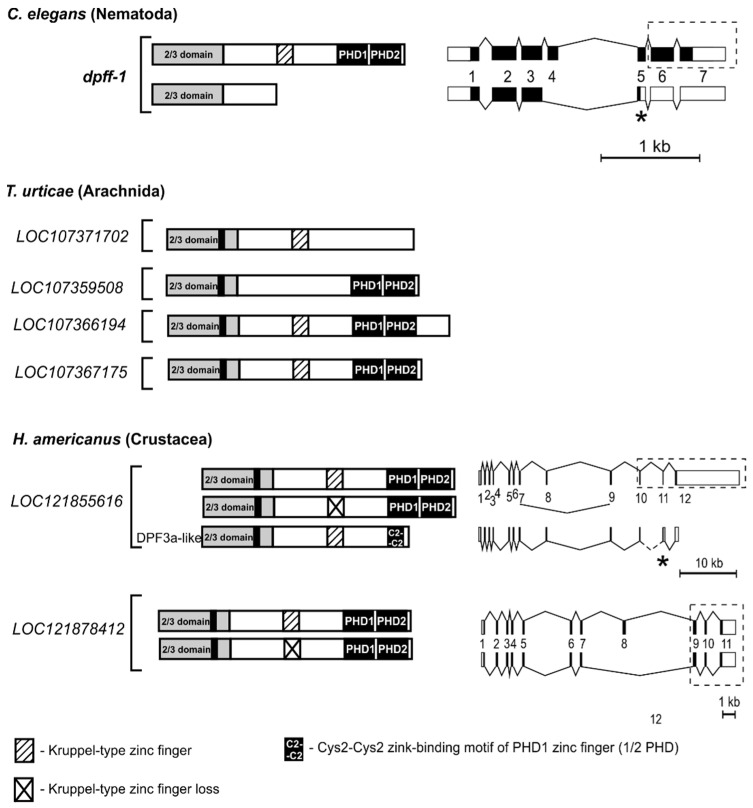
Domain organization of the protein products of the *d4* family genes of some Nematoda, Arachnida, and Crustacea species. The 2/3 domain is filled with gray, and the black bar indicates NLS. The PHD1 and PHD2 zinc fingers of the D4 domain are shown as black boxes. On the left are schemes of alternative splicing, showing positions of exons (vertical lines) and scale bars. The exons coding for the D4 domain are in dashed boxes. The asterisk “*” indicates splicing causing a frameshift.

It turned out that one of the *d4* paralogs in spider mite *T. urticae* lacks exons coding for the D4 domain (Figure 4).

We also found that one of the *d4* paralogs in spider mites *T. urticae* and *P. citri* and in the crustacean species *E. affinis* does not encode the Kruppel-type ZF. So far, only the Diptera *d4*-related genes have been found to have such specific features (Table 2).

In deuterostomes, the first duplication of the *d4* family gene occurred in the sea lamprey (*P. marinus*) (Table 3 and Table S1). Three *d4* family genes were found in the cartilaginous fish *Carcharodon carcharias* and other vertebrates, except bony fishes (class Actinopterygii) in whose genomes multiple (four or more) copies of *d4* genes have been found (Table S1). It turned out that in some bony fishes (*Oryzias melastigma*, *Hypomesus transpacificus*, *Pangasianodon hypophthalmus*, and *Gambusia affinis*) one of the *d4* family paralogs has a loss of the last exons encoding the D4 domain and thus encodes the DPF3a-like protein (Table 3 and Table S1).

Thus, we conclude that the divergence of duplicated *d4* genes in protostomes (spider mites, Diptera species) and deuterostomes (some bony fish) may have resulted in the loss of the D4 domain coding sequence.

The next step was to analyze the domain organization of the isoforms produced by *d4* family genes.

### 2.4. The Domain Organization of the Isoforms Produced by the d4-Related Genes

A preliminary analysis of the protein database allowed us to suggest that the evolutionary path of the *d4* family genes is characterized by neofunctionalization, expressed in the appearance of isoforms with different domain organization. Therefore, we performed a more detailed taxon-focused search in the NCBI RefSeq database for predicted protein isoforms.

#### 2.4.1. Isoforms Lacking Kruppel-Type ZF

A consensus sequence Cys(X)2Cys(X)12His(X)4His of the Kruppel-type ZF was derived from an alignment of D4 proteins from 14 animal species belonging to different taxonomic groups, from Placozoa to Chordata (Figure S8). The first isoform of D4 protein we found in non-bilaterian, in the sea anemone *Nematostella vectensis.* In addition to the full-length protein, the *N. vectensis d4* gene produces an isoform that lacks the Kruppel-type ZF, which is the result of an exon-skipping splicing. Similarly, organized protein isoforms were then found in many protostomes (Table 2). In deuterostomes, isoforms lacking Kruppel-type ZFs have been found in invertebrates, for example, in the starfish *A. rubens* (Echinodermata) (Table 2). As in protostomes, this predicted isoform can be generated via exon-skipping splicing. In vertebrates, we did not find isoforms lacking Kruppel-type ZF. However, the C2H2 zinc finger structure in some isoforms appears to be disrupted by a 14 a.a. insertion between a pair of Cys residues as a result of exon inclusion splicing (Table 3, Figure 3 and Figure S5D). This insertion has high sequence conservation in vertebrates, suggesting that it is necessary for the new function of such disrupted Kruppel-type ZFs in certain isoforms.

#### 2.4.2. Isoforms Lacking the D4 Domain

The first isoform lacking the D4 domain was found in the protostomes among the protein products of a single-copy *d4* family gene in nematode *C. elegans* (Table 2). As a result of exon-skipping splicing causing a frameshift in the D4 domain coding sequence, only the 2/3 domain is present in this C-terminally truncated protein isoform (Figure 4). Further analysis of protostomian *d4* genes reveals the presence of DPF3a-like isoforms in the NCBI gene models of insects (described above) and crustaceans *Penaeus japonicus*, *Homarus americanus*, and *Portunus trituberculatus* (Table 2). It is noteworthy that in these crustaceans, predicted DPF3a-like isoforms can be found among the protein products of only one of the two *d4* paralogs, which may be an indication of the specialization of the genes in their ability to produce these isoforms. According to the NCBI gene models, the DPF3a-like isoforms of these crustaceans are generated by alternative splicing causing a frameshift in the D4 domain coding sequence (Figure 4).

The analysis of protein isoforms from the NCBI *d4* family gene models of deuterostomes from the phylum Echinodermata, Hemichordata, and ancient chordates (Ascidiae and Lancelets) that possess single-copy genes reveals no isoforms lacking the D4 domain. We have discovered that according to the NCBI databases, the evolutionary first DPF3a isoform of deuterostomes emerged in the jawless cartilaginous fish, *Petromyzon marinus*. This fish’s genome has a duplicated *d4* gene, and one of the copies is responsible for producing this specific variant. The analysis of other vertebrate genomes has revealed that as in sea lamprey, only one of several *d4* paralogs is capable of producing the DPF3a isoform in bony and cartilaginous (chondrichthyan) fish, amphibians, reptiles, birds, and mammals (Table 3).

We have found that these isoforms may arise by alternative splicing via the mechanism of the alternative last exon inclusion (ALE). Furthermore, we have noted that the C-termini (XZ) of these isoforms display a high degree of conservation (Figure S6C). In vertebrates, the only example of an isoform other than the DPF3a type, which lacks all the structural elements of the D4 domain, was found among the products of one of the paralogs of the *d4* family genes in zebrafish (Table 3).

Thus, we found that the most common isoform lacking the D4 domain generated by the *d4* family genes is DPF3a in vertebrates and DPF3a-like in invertebrates.

#### 2.4.3. Evolutionary Conservation of 2/3 Domain

The 2/3 domain was previously identified as the N-terminal conserved sequence of the *d4* family proteins, encoded by exons 2 and 3 (52 a.a. and 35 a.a., respectively) in the mammalian neuro-d4, ubi-d4/Requiem, and Cer-d4 genes [5,6]. The multiple alignment of the 14 D4 family protein sequences from species of different taxonomic groups (placodes, sponges, cnidaria, molluscs, nematodes, arachnids, crustaceans, insects, echinoderms, hemichordata, and chordata) revealed that this conserved domain sequence is located within the region spanning 68–71 a.a. (Figure S8). However, the interspecies comparison of proteins within the same taxonomic group (class and order) shows that the length of the conserved 2/3 domain sequence in proteins from different animal taxonomic groups may be longer than 68–71 a.a. and extended N-terminally along this core conserved region and this may reflect the evolutionary divergence of 2/3 domains in *d4* family orthologs.

#### 2.4.4. The 2/3 Domain Amino Acid Sequence Divergence in *d4* Paralogs

Since we have found in Diptera that the sequence divergence between the 2/3 domains of *tth*-like and *dd4*-like paralogs in the same species is greater than between their counterparts in different species, we wondered whether such a pattern of divergence would also be present in the *d4* family paralogs of other taxa. For our analysis, we chose the *d4* paralogs from crustaceans and vertebrates because they have a sufficient number of annotated genomes in the NCBI database (Table 2, Table 3 and Table S1). The amino acid sequences of 2/3 domains from the protein products of *d4* family genes from five crustacean species having two paralogs *H. azteca*, *P. japonicus*, *H. americanus*, *P. trituberculatus*, and *E. affinis* (Table 2) were used for alignment. The alignment revealed the clustering of the paralogs by two groups with higher sequence similarity. The first group having 57.61–97.83% identity between the sequences of the 2/3 domains includes *H. azteca LOC108677705*, *P. japonicus LOC122250070*, *H. americanus LOC121855616*, and *P. trituberculatus LOC123508560 genes*. The second group includes *H azteca LOC108672214*, *P. japonicus LOC122250696*, *H. americanus LOC121878412*, and *P. trituberculatus LOC123516819* genes, which have 64.52-90.32% identity between the sequences of the 2/3 domains. The intergroup similarity between the sequences of the 2/3 domains of the paralogs possesses 40.22–51.09% identity (Figure S4B). It is interesting to note that the first group of paralogs includes genes that have predicted DPF3a-like isoforms in the protein products (Table 2), which is obviously reflected in the level of divergence of the paralogs in crustaceans. The *d4* paralogs from *E. affinis* have not been referred to any group because of the lowest sequence similarity between their 2/3 domains and the domains from other crustaceans (28.26–40.86% identity), possibly due to the species being phylogenetically remote from the other crustaceans analyzed (Figure S4B).

Three DPF1-DPF3 paralogs of human, mouse, chicken, lizard, frog, and shark (*Homo sapiens*, *Mus musculus*, *Gallus gallus*, *Lacerta agilis*, *Rana temporaria*, and *Carcharodon carcharias*, respectively), four paralogs of zebrafish, and two paralogs of sea lamprey were used to analyze the evolutionary sequence divergence of the 2/3 domains of vertebrates (Figure S4C). A sequence similarity analysis showed that the similarity between the 2/3 domains in paralogs from the same species is lower than that between their respective counterparts from different species. Thus, we concluded that the divergence pattern of the 2/3 domain sequences in the dipteran, crustacean, and vertebrate *d4* paralogs may reflect their functional divergence.

#### 2.4.5. Protein Isoforms with Truncated or Missing 2/3 Domain

The ability of some paralogs to generate isoforms with a missing or truncated 2/3 domain may also be one of the characteristics of paralog divergence. In protostomes, the D4 protein isoforms lacking the 2/3 domain were found in the protein products of *dd4*-like genes from three Drosophila species (*D. melanogaster*, *D. suzuki*, and *D. eugracilis*), but not in *tth*-like genes (Figure 1A and Figure S1C, Table 2).

In deuterostomes, the isoforms with an altered 2/3 domain are found in the products of the vertebrate *Dpf1* gene. In contrast to *Drosophila*, the vertebrate N-terminally truncated isoforms have partially retained the 2/3 domain due to alternative transcription and a specific splicing pattern of the *Dpf1* gene (Figure 3, Table 3). The vertebrate 2/3 domain is encoded by exons 2 and 3, but some transcripts generated from alternative promoters have a truncated coding region of exon 2, while the NLS-containing sequence of the 2/3 domain encoded by exon 3 is retained (Figure S5D). However, we found that one of the isoforms in the human *Dpf1* gene, due to alternative transcription started downstream of exon 3, completely lacks the sequence of the 2/3 domain (Table 3). We also found isoforms with a truncated 2/3 domain among the predicted protein products of the *Dpf3* gene in the reptile *Lacerta agilis* and in aves *Gallus gallus*. This indicates that such isoforms are not unique for *Dpf1* (Table 3). Finally, we found no isoforms with a missing or truncated 2/3 domain in the predicted protein products of single-copy *d4* family genes from non-bilaterian, protostomian, and deuterostomian species.

### 2.5. The Results of the Phylogenetic Analysis Allowed Us to Draw the Following Conclusions

1. Single-copy genes always encode a 2/3 domain and a D4 domain.
2. Non-bilaterian animals have a single copy of the *d4* family gene, which encodes a protein with all the characteristic domains and is capable of producing the full-length protein and the only variant of isoforms lacking the Kruppel-type ZF.
3. Protostomes may have one or more copies of the *d4* family genes. The single-copy genes can produce full-length proteins and isoforms lacking either Kruppel-type ZF or the D4 domain. For multiple-copy genes, the ability to generate isoforms lacking the D4 domain is characteristic of one of the paralogs.
4. Like protostomes, deuterostomes may have one (hemichordates, lancelets, ascidians, and echinoderms) or more (vertebrates) copies of the *d4* family genes. In vertebrates, a wide variety of isoforms was found: variants with a 2/3 truncated domain, variants with Kruppel ZF disruption, and variants lacking a D4 domain, and each of the paralogs is capable of generating its own specific spectrum of isoforms. However, in deuterostomes with a single copy of the *d4* gene, in contrast to protostomes, no isoforms lacking the D4 domain have been found.
5. The most common isoform of D4 family proteins lacking the D4 domain is DPF3a in vertebrates and DPF3a-like in invertebrates.
6. Finally, we found no *d4* family genes that do not encode the 2/3 domain, but in both protostomes and deuterostomes, we found *d4*-related paralogs encoding proteins lacking the D4 domain. Therefore, the unique 2/3 domain coding region can currently be considered as a hallmark of the genes in this family.

## 3. Discussion

Two paralogs of the *d4* family, *dd4* and *tth*, which were previously found in *Drosophila* [17,18], both encode proteins with a conserved N-terminal 2/3 domain but lack a central Kruppel-type ZF. However, they show considerable structural disparities in the C-terminal region. DD4 contains a C-terminal D4 domain, whereas TTH lacks it. The bioinformatic analysis of insect genomes performed in our study revealed that only Diptera possess *tth* and *dd4* paralogs. An exception is the mosquito species of the Anopheles genus, in which a single *dd4*-like gene has been identified. Single *d4* gene copies are present in the genomes of other insects as well. However, they differ from *dd4*-like orthologs since they encode all the typical domains of the D4 family (Figure 3). The presence of the 2/3 domain in the protein products of *tth*-like and *dd4*-like genes suggests that these genes could be related and might have been formed due to duplication and subsequent divergence from the ancestral *d4* gene. Duplicated gene copies may either retain the original function of the ancestral gene or diverge, acquiring a new one [27]. To investigate possible divergence pathways for duplicated *d4* gene copies and to identify the evolutionary traces of ancestral genes, we reconstructed the evolutionary history of the *d4* family genes.

**Figure 5:**
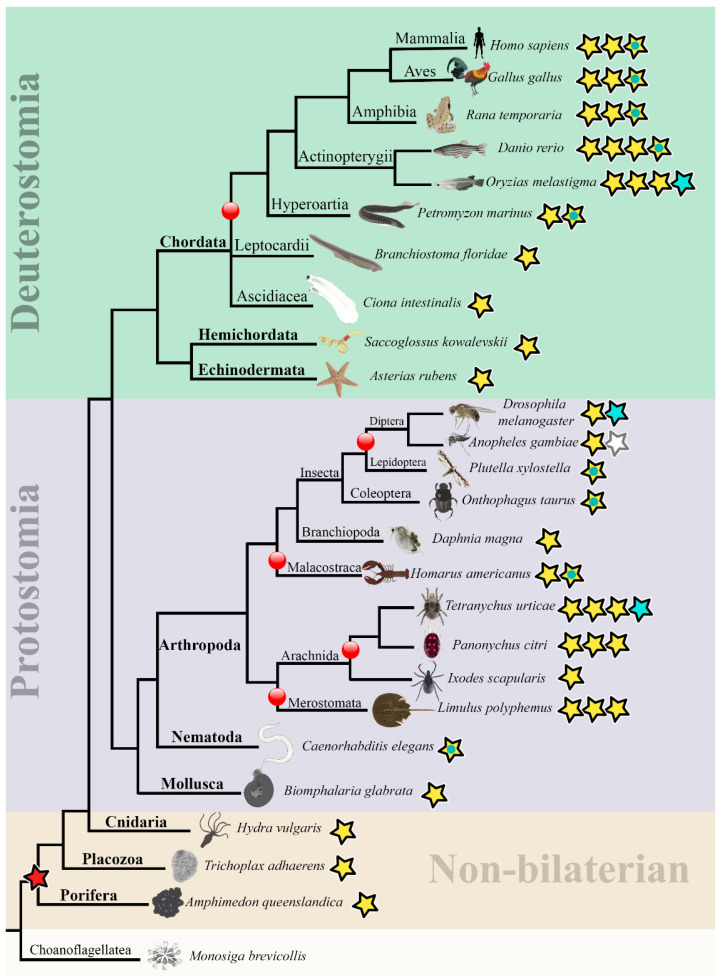
The reconstructed evolutionary history of *d4* family genes in the Animalia kingdom. A phylogenetic tree schematically illustrates the expansion of *d4* family genes. The appearance of the first *d4* gene is marked by the red star symbol. The red circles mark the duplication events. The number and color of stars indicate the number of genes encoding proteins that contain (yellow) or lack (blue) the D4 domain in the selected species. The white star indicates a gene loss event. If a yellow star has a blue dot inside, it indicates that the gene encodes an additional isoform lacking the D4 domain.

The evolution of the D4 family genes. To identify *d4*-related genes, we conducted a genome survey of various taxonomic groups, including protists, non-bilaterian organisms, protostomes, and deuterostomes. Our analysis revealed the presence of the orthologs of *d4* in metazoans, but not in protists (Figure 5). We made the conclusion that non-bilaterians (Porifera, Placozoa, and Cnidaria) and protostome mollusks and worms (Nematoda) possess a single gene related to the *d4* family, while in Arthropoda, single and multiple copies of the *d4*-related genes were identified in various species (Figure 5, Table 1 and Table S1). In deuterostomes, a single copy of the *d4* family gene was found in species belonging to Echinodermata, Hemichordata, and also in chordates in Ascidia and Lancelet, and the first duplications occurred in the evolution of vertebrates: we found two *d4* genes in cyclostomes, four or more in bony fishes, and three genes in other vertebrates, including humans (Figure 5, Table 1 and Table S1). Therefore, it can be inferred that the duplications of the *d4* family genes occurred during the parallel evolution of protostomes and deuterostomes in arthropods and vertebrates, respectively.

The analysis of domain organization of the D4 family proteins. Previous studies have shown that mammalian *d4* family genes have complex splicing patterns, resulting in isoforms with different domain organization [2,5,6]. Since then, a considerable amount of genomic and transcriptomic data has been accumulated. In this work, we analyzed these data to identify trends in the evolution of D4 family genes associated with their ability to produce protein isoforms and proteins with different domain organizations.

D4 family proteins lacking a Kruppel-type zinc finger. Our research has revealed that the *d4* genes in all animals, except Diptera *tth* and *dd4*, along with certain paralogs of spider mites *T. urticae* and *P. citri*, contain exons that encode a Kruppel-type zinc finger (Table 2). However, a search on the NCBI protein database revealed that *d4* genes could generate a splice isoform with a disrupted or missing ZF. Evolutionarily, the first isoforms lacking the Kruppel-type ZF appear in non-bilaterians, and in coelenterates (sea anemones). Then, they could be found in species of all protostome taxa, having either one or more *d4* gene copies (Table 2). The D4 isoforms of non-bilaterians and protostomes lack the Kruppel-type ZF as a result of exon-skipping splicing. The same splicing type generates a D4 isoform lacking Kruppel-type ZFs in the deuterostome invertebrate starfish *A. rubens* (Echinodermata) (Table 2). In vertebrates, however, the splicing pattern that affects the structure of the Kruppel-type ZF is different and proceeds through the exon inclusion, resulting in 14 a.a. insertion between the first pair of Cys residues (Figure 3 and Figure S5D). The C2H2 ZF motif has been classified as Kruppel-type based on the specific combination of zinc-binding a.a. similar to those that form DNA-binding ZF clusters in transcription factors belonging to the Kruppel family [2]. However, the D4 proteins have no DNA-binding properties [14], and their single Kruppel-type ZF shares a remarkably high homology with the C-terminal ZF of the yeast Split Finger Protein 1 (SFP1) (Table S1 of [7]). It is known that this protein plays a key role in ribosome biogenesis, regulates G2/M transitions during the mitotic cell cycle, and its C2H2 ZFs are responsible for the protein–protein interaction with the nuclear factor Mrs6, which targets it to the nucleus [28,29,30]. The crystal structure of the C2H2 Kruppel-type ZF DPF2 of human DPF2 revealed structural features, such as conserved positively charged amino acids and a hydrophobic core, characteristic of the ZF of the proteins ZNF268, ZNF484, and NEMO, which are involved in both DNA and protein interactions [25]. Since no RNA-/DNA-binding properties have been shown for the Sfp1-like Kruppel-type ZF in D4 proteins, we can speculate that, as in yeast, this zinc finger is also responsible for protein–protein interaction in metazoans. The existence of D4 family proteins lacking the Kruppel-type ZF may indicate that this ZF is not essential for the function of certain D4 protein isoforms. This may suggest an evolutionary drift toward the specialization of *d4* paralogs, as in mice, where only one of three paralogs, *Dpf2*, can produce specific isoforms with disrupted Kruppel-like ZFs. Another example can be seen in *T. urticae* and *P. citri* spider mites, where one of the *d4* paralogs lacks Kruppel-type ZF coding sequence (Table 2, Figure 4).

Finally, both *tth*-like and *dd4*-like paralogs in Diptera do not encode the Kruppel-like ZF, and therefore, the function that could be associated with this motif appears to be completely abolished in dipteran D4-related proteins.

The D4 family proteins that lack the D4 domain. Our research demonstrates that *d4* genes from various animals are capable of producing isoforms that lack the D4 domain. The most evolutionarily ancient animal in which we found such isoform was the nematode *C. elegans*. The appearance of an isoform lacking the D4 domain at an early stage in animal evolution indicates that this protein product is essential for the organism. It is, therefore, reasonable to conclude that all animals in evolutionary hierarchies above nematodes may possess such protein products. Our analysis of the NCBI data shows distinct C-terminally truncated D4 protein isoforms: (1) the isoform truncated downstream of the 2/3 domain found in *C. elegans*, (2) the isoform truncated downstream of the Kruppel-type ZF found in *F. occidentalis* (Insects/Thysanoptera), (3) the DPF3a isoforms found in vertebrates and DPF3a-like isoforms found in arthropods are truncated downstream of the exon encoding the C2C2 motif of PHD1 of the D4 domain (Table 2, Figure 3 and Figure 4).

The *d4* family genes, which are capable of producing DPF3a/DPF3a-like isoforms have three-four exonic structures encoding the D4 domain, and the donor splice site of the exon encoding the C2C2 motif of PHD1 in the D4 domain is a conserved splicing “hotspot”, facilitating the generation of such isoforms. It is notable that this splice site is conserved even in non-bilaterians, suggesting that the capability to produce DPF3a-like isoforms may have arisen before animals split into two main groups: protostomes and deuterostomes.

It is also important to note that in contrast to vertebrates, which have a common alternative last exon type of splicing pattern for the generation of DPF3a isoforms, the DPF3a-like isoforms in arthropods are generated through different splicing mechanisms. In decapod crustaceans, this occurs via using an alternative acceptor splice site, which causes a frameshift and premature termination codon, while in insects, two primary mechanisms are involved: exon extension and ATT, as well as alternative last exon splicing (Figure 3 and Figure 4). This suggests that the evolution of similarly organized isoforms may have followed different paths.

The distinguishing feature of *d4* genes with the ability to produce DPF3a-like isoforms is a considerable span of the gene locus, encompassing the D4 domain exons (Figure 3 and Figure 4).

It is interesting that in animals with two or more *d4* copies, we found that only one of the paralogs is capable of producing DPF3a-like isoforms. For example, among the protostomes, in the decapod crustaceans *H. americanus*, *P. japonicus*, and *P. trituberculatus*, which have two *d4* paralogs, DPF3a-like isoforms were found for only one of them (Table 2). Among deuterostomes, which include mammals, birds, reptiles, and amphibians with three, and fish with four or more *d4* paralogs, only one of the paralogs has the capacity to produce the DPF3a isoform (Figure 5, Table 3), which reflects the tendency to specialize one of the paralogs for the maintenance of a new function. Finally, among the paralogs of the *d4* family in protostomes and deuterostomes, we found examples of the further specialization of one of the paralogs to encode the only protein with loss of the D4 domain. In protostomes, one such gene was identified among four paralogs of the *d4* family in the spider mite *T. urticae*. Another instance is the *tth*-like paralog in Diptera (Table 2). In deuterostomes, we found a similar example of gene specialization in the fish *O. melastigma*, *H. transpacificus*, *P. hypophthalmus*, and *G. affinis* (Table S1, Figure S6C). The evolution of the *Dpf3* paralog in these fish resulted in the loss of some exons coding for the D4 domain of the full-length protein, while the coding structure of the DPF3a isoform was retained. The residual C2C2 motif of PHD1 in these proteins has undergone significant sequence divergence (see Figure S6C). The preceding examples indicate the potential for the structural divergence of the D4 family genes, resulting in the loss of exons encoding the D4 domain in one of the paralogs. It is also important to note that no loss of exons encoding the D4 domain was observed in the evolution of single-copy D4 family genes.

We were wondering about the significance of the structural divergence related to the loss of the D4 coding region. It has been demonstrated that mammalian D4 family proteins (BAF45b-d) are subunits of the SWI/SNF-like ATP-dependent chromatin remodeling BAF complex [8,9] and the D4 domain is responsible for binding to acetylated and methylated lysines on histones H3 and H4 and thus functions as a histone reader [7]. Since the other subunits of the complex lack PHD fingers, the targeting/recruiting of the BAF complex to specific histone marks may be the primary function of the D4 proteins (BAF45b-d) in the BAF complex [9]. However, the splice isoform DPF3a, which lacks the D4 domain, may recruit the BAF complex to other targets such as the HEY repressor [14] or the epigenetic reader HRP2 [15], indicating its functional divergence towards the regulation of specific genes. The C-terminal sequence of DPF3a isoform possesses high similarity among species of all classes of vertebrates (Figure S6), suggesting the functional similarity of DPF3a in mammals, birds, reptiles, amphibians, fish, and cyclostomes. The phosphorylation of DPF3a by casein kinase II is required for DPF3a-HEY binding, and the phosphorylation site is conserved in human, mouse, and chicken DPF3a [14], while the IBD-binding motif (IBM) for HRP2 binding found in mouse DPF3a [15] is characteristic of all vertebrate DPF3a proteins (Figure S6). In contrast to vertebrates, the insect DPF3a-like isoforms have distinct C-tail sequences that are homologous exclusively between species within the same order (LXZ, HXZ, or CXZ tails) (Figure S6), indicating that their C-termini possess distinct taxon-specific functions. Not only variations in the structure of the C-terminus of DPF3a-like isoforms, but also the different types of alternative splicing that generate DPF3a-like isoforms reveal the significant evolutionary divergence of insect *d4* genes (Figure 3C and Figure S5).

The C-terminal conserved region of TTH-like paralogs (Figure 1A and Figure S1A), designated here as C-tth, is also unique to Diptera. We have proposed that this motif is likely to be inherited from an isoform lacking a D4 domain (which may be similar to DPF3a), a product of the single-copy ancestral gene. Furthermore, we proposed that the Drosophila *tth* and *dd4* paralogues share the domain organization of different isoforms originating from a single gene of the Diptera ancestor. These assumptions were deduced from an analysis of the evolutionary history of *d4* genes in insects.

The evolution of *d4* family genes in insects—Why Anopheles is different? In this study, we found that only Diptera among insects has two paralogs, *tth*-like and *dd4*-like, except *Anopheles*, which has a single *d4*-related gene (Table 2 and Table S1), sharing the same features as the *dd4*-like genes, i.e., it does not encode for a Kruppel-type ZF. Mosquitoes diverged from other Diptera approximately 260 million years ago (MYA), while Anopheles diverged from other mosquitoes approximately 150 MYA (Figure 1B). It can, thus, be proposed that the *Anopheles dd4*-like gene may be in the ancestral single-copy state. However, it is also possible that during evolution, one of the two copies has been lost.

The evolutionary history of *dd4* and *tth* paralogs in Diptera inferred by using the Maximum Likelihood method revealed that the *dd4* single gene state of *Anopheles* mosquitoes highly likely results from the loss of one of the copies (Figure S2A). It has been reported that in *Anopheles*, X-chromosome rearrangements are 2.7 times more frequent than autosomal rearrangements. The rate of gene gain/loss is at least five times higher than in *Drosophila* [22]. Our analysis of the distribution orthologs of *Aedes* and *Culex tth* anchor genes across the *Anopheles* X chromosome has led us to the conclusion that the *tth* ancestral gene of *Anopheles* may have been either deleted or disrupted by multiple transposon insertions (Figure S2B).

**Figure 6:**
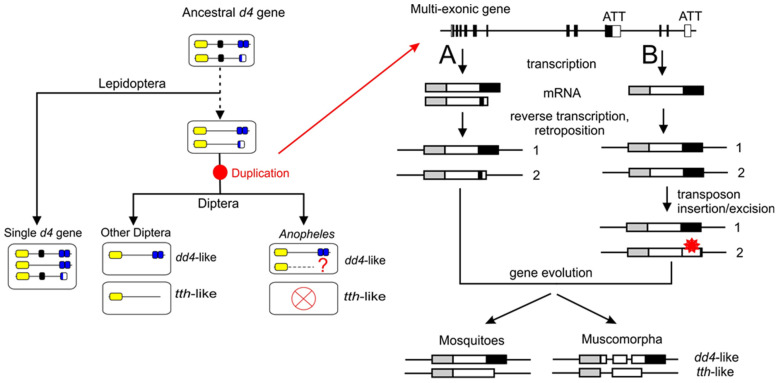
A hypothetical scheme for the evolution of *d4* family genes in insects. The diagram on the left illustrates the proposed evolutionary pathway of the *d4* family genes in insects. The schematic domain organizations of gene protein products are shown inside boxes. Yellow, domain 2/3; blue, D4 domain; black, Kruppel-type zinc finger; blue-white, C-terminus of DPF3a-like isoform. Note that *tth*-like gene is eliminated in the genomes of the genus *Anopheles* (red crossed circle). The potential scenarios for the *d4* gene duplication in the order Diptera, pointing by the red arrow, are illustrated on the right (A and B). Gray boxes represent the 2/3 domain coding regions. Regions that code for the D4 domain or a part of the domain are shown as black boxes. The red star indicates a disruption in the domain coding region. Designations: ATT—alternative transcription termination site; 1, 2—gene copies.

We hypothesize the following pattern of evolution for *d4* family genes in insects: First, the ancestral *d4* gene for Lepidoptera/butterflies and Diptera encoded all the characteristic domains and produced at least two protein isoforms with or without the D4 domain. During evolution, butterflies acquired this gene unchanged, whereas in the common ancestor of Diptera, the region encoding the Kruppel-type ZF was lost, followed by the duplication of an altered gene (Figure 6, left side). An analysis of gene structure for *tth*-like and *dd4*-like orthologs led us to the conclusion that the duplication of *d4* family genes in the Diptera order is most likely the result of retroposition events. This is consistent with the intronless structure of the coding regions in mosquito paralogs and the intron-poor structure specific to each of the paralogs in other Diptera. Nevertheless, one paralog should exhibit the characteristics of the ancestral single-copy gene, such as a comparable exon/intron structure. The single-copy *d4* genes of insects closely related to Diptera are characterized by a multiexon structure (Figure S5) and a substantial number of intron positions overlap between orthologs. The absence of a paralog of a similar structure in Diptera may suggest that the original gene has been lost during evolution, possibly due to potential functional redundancy. For example, the original gene loss with the retention of a retrocopy has been described in mammals and fish [31,32].

We have put forth two potential scenarios for the duplication of the original gene. The first scenario is based on the specific splicing pattern of the *d4* genes of insects that are closely related to Diptera. These genes have the capacity to generate two distinct transcripts, one encoding a full-length protein and the other a truncated DPF3a-like isoform. The transcripts are terminated at alternative transcription termination sites (ATT). We proposed that the *d4* gene in the Diptera ancestor exhibited a similar pattern of generation for the alternative transcripts, and that these two transcripts may have been subject to retrocopying. In this instance, retroposed genes exhibited a similar coding structure to that of the isoforms produced by the original gene (see path A in Figure 6, right side).

In another scenario, the only transcript encoding the full-length protein may have been subject to retrocopying. In this instance, both retrocopies exhibited a near-identical structural framework (see path B in Figure 6, right side). It is possible that one of the duplicates subsequently may have either lost or disrupted the D4 domain coding region as a result of locus rearrangement, potentially by a transposon insertion/excision event. This gene may evolve to replace the function of the isoform lacking the D4 domain produced by the original gene. In considering these two hypothetical scenarios, we suggest that the resulting paralogs, *tth*-like and *dd4*-like, may have undergone subfunctionalization to share the function of the ancestral single-copy gene.

Next, during dipteran evolution, the *tth*-like paralog was lost in *Anopheles*, while the other dipterans retained two paralogs, *tth*-like and *dd4*-like (Figure 6, left side). Notably, in contrast to Lepidoptera, we did not find any isoforms lacking the D4 domain among the predicted protein products of single-copy *dd4*-like genes in *Anopheles*. Perhaps these isoforms exist but are difficult to predict and therefore, not currently annotated. It should be noted that according to the NCBI gene models, the *dd4*-like genes in *Anopheles* mosquitoes have an intronless coding sequence. However, one of the annotations for the *dd4*-like gene in *An. gambiae* suggested a three-exonic structure with two in-frame introns, which was supported by the TSA data (Figure S1C). This suggests that *Anopheles dd4*-like genes may have cryptic introns, and therefore, the generation of isoforms with the loss of the D4 domain by alternative splicing leading to a frameshift is highly likely. We believe that future targeted analysis of real transcripts may clarify this issue.

The sequence divergence and functional implication of 2/3 domain. The evolutionary history of the D4 family of genes demonstrates that paralogs lacking Kruppel-type ZF or D4 domain-coding exons can emerge in different evolutionary lineages. In single-copy genes, the loss of Kruppel-type ZF coding sequence was observed exclusively in *Anopheles*. However, no examples of genes lacking the 2/3 domain coding sequence have been found among either single-copy genes or paralogs. Thus, we concluded that the 2/3 domain is the primary and defining feature of the family.

In the animals with a single copy of the *d4* family gene, no protein isoforms with a truncated or missing 2/3 domain were found. However, in animals having *d4* paralogs, one of the genes can generate isoforms with the truncation or loss of the 2/3 domain, as is the case for the protein products of vertebrate neuro-*d4/Dpf1* and *Drosophila dd4* genes (Figure 3). These isoform variations in D4 family proteins may modulate their function by reducing the spectrum of protein partners interacting with them. On the other hand, the sequence divergence between 2/3 domains that we found in vertebrate, crustacean, and dipteran D4 paralogs (Figure S4) may indicate a neofunctionalization of these domains associated with specialization for interactions with different target proteins. For example, it has been shown that DPF2 and DPF3 proteins are involved in the different NF-κB pathways by interacting with the RelB/p52 heterodimer of non-canonical and the RelA/p50 heterodimer of the canonical pathway, respectively [12,13]. A key role in this interaction has been shown for the NLS-containing N-terminal region of human REQ/DPF2 (domain 2/3), which directly binds subunits of the BAF complex and the RelB/p52 heterodimer [12], so it is conceivable that sequence divergence between the domains 2/3 of D4 paralogs may reflect their interaction with different NF-κB factors. Interestingly, in *C. elegans*, which lacks NF-κB transcription factors [33], the protein product of the single-copy *d4* family gene exhibits the lowest conservation in the 2/3 domain sequence (Figure S8). This suggests that the sequence conservation of domain 2/3 is maintained in evolution due to its dual function in interacting with SWI/SNF-like BAF complex and NF-κB transcription factors. The sequence divergence of the 2/3 domains detected in *Drosophila* TTH and DD4 proteins (Figure 1A) also may determine the specificity of the domains to target proteins. Like their mammalian counterparts, the *Drosophila* TTH and DD4 proteins were found to be associated with the SWI/SNF-like BAP chromatin remodeling complex [9,19]. Given that mammalian D4 proteins participate in the NF-κB pathway through their 2/3 domain which interacts with the SWI/SNF chromatin remodeling complex and different NF-κB heterodimers, we hypothesized that the 2/3 domain of *Drosophila* TTH and DD4 proteins might interact with the fly NF-κB family homologs—Dorsal, Dif, and Relish in a similar manner. Thus, the differential involvement of the 2/3 domain of *tth*-like and *dd4*-like proteins in the NF-κB pathway can be studied in the *Drosophila* model. It should be noted that in the N-terminal halves of D4 family proteins, there is a region that is not associated with the 2/3 domain that is highly related between them. This includes Lys-Arg repeats (in some proteins, these contain predicted NLS2) and adjacent upstream sequences (Figure 1A and Figures S1A,B, S7 and S8). These sequence motifs are constantly present in all splice isoforms, either N-terminally or C-terminally truncated. Given that these sequences are a part of the R2 region, which has been demonstrated to interact with subunits of the BAF/BAP complex, in addition to the 2/3 domain (Requiem) [9], it is reasonable to propose that this region enables isoforms lacking the 2/3 domain to interact with a SWI/SNF-like complex.

## 4. Materials and Methods

### 4.1. Bioinformatics

#### 4.1.1. Identification of *d4* Family Orthologs and Paralogs

To search for *Drosophila melanogaster tth* (*toothrin*, CG12175, FBgn0030502) and *dd4* (*d4*, CG2682, FBgn0033015) orthologs and the orthologs of the mammalian *d4* family genes: *neuro-d4*, *ubi-d4*, and *cer-d4* (Dpf1-3), we used the “OrthoDB” (OrthoDB v11 [21], www.orthodb.org, accessed on 3 March 2024) and “NCBI Orthologs”, databases available in the NCBI Gene Annotation Resources (https://www.ncbi.nlm.nih.gov/gene/, accessed on 29 March 2024). In order to distinguish the *d4* family genes from the genes that are occasionally classified as *d4* orthologs, we reviewed the domain content of the encoded proteins provided by NCBI Entrez Gene. Additionally, taxon-focused searches were conducted to identify gene copy numbers and enhance the comprehension of gene evolution from Placozoa to Chordata. To this end, a search for similar sequences to the query sequence was conducted in the protein and nucleotide databases of the National Center for Biotechnology Information using the BLAST tool (https://blast.ncbi.nlm.nih.gov/Blast.cgi, accessed on 10 September 2024) [34]. Using the amino acid sequences of the conserved domains of D4 family proteins (domain 2/3 and D4 domain) as BLAST query in BLAStP search [35,36], we searched the NCBI RefSec protein database for isoforms in the protein products of *d4* orthologous and paralogous genes from the selected taxa. A list of identified *d4* family genes from 121 species belonging to various evolutionary lineages is provided in Table S1. To demonstrate the evolutionary relationships between species, phylogenetic trees were constructed using the TimeTree resource (http://timetree.org, accessed on 15 October 2024) [37].

The multiple sequence alignment diagrams were generated using the COBALT alignment tool [38] from the NCBI Alignment Service Tools (https://www.ncbi.nlm.nih.gov/tools/cobalt/cobalt.cgi, accessed on 12 June 2024) and BioEdit 7.2. sequence alignment editor.

For the prediction of nuclear localization signals (NLSs), the protein subcellular localization prediction tools PSORT II (https://www.genscript.com/psort.html, accessed on 10 May 2024) [39] and DeepLoc 2.0 (https://services.healthtech.dtu.dk/services/DeepLoc-2.0/, accessed on 10 May 2024) [40] were used.

#### 4.1.2. Isoforms Validation

To verify the predicted isoforms identified in the gene models, we conducted a BLAST search of the Transcriptome Shotgun Assembly (TSA) database using the TBLASTN algorithm [36] with isoform-specific sequences as the query. The deduced amino acid sequences of the ORFs of TSA transcripts were obtained using SnapGene Viewer 5.1 (the SnapGene^®^ software from Dotmatics (Boston, MA, USA), available at https://www.snapgene.com/, accessed on 20 September 2024), and aligned to the predicted NCBI RefSeq protein isoform sequences. A multiple sequence alignment was conducted using the Clustal Omega program [41], which is accessible via the Uniprot website (https://www.uniprot.org, accessed on 20 September 2024). The NCBI RefSeq protein isoforms were confirmed in two ways: directly by similarity to protein sequences obtained from TSA transcripts from the same species, and indirectly by establishing homology with protein sequences obtained from TSA transcripts from related species belonging to the same order.

#### 4.1.3. A Comparative Analysis of the Sequence Divergence Between Domains of the *d4* Paralogs

The evolutionary sequence divergence between the 2/3 domains of the protein products of the *d4* family gene paralogs has been studied in Diptera (insects), crustaceans, and various vertebrate species for which sufficient data were available in the NCBI database. The amino acid sequences of paralogous proteins were retrieved from the gene models and aligned for each taxonomic group separately using the Clustal Omega software (version 1.2.4). The conserved sequences belonging to the 2/3 domains, identified through alignment, were retrieved from the full-length proteins and aligned to generate a percent identity matrix and a guide tree dendrogram, which show groups of closely related sequences (Figure S4).

#### 4.1.4. Phylogenetic Analysis by Maximum Likelihood Method

Evolutionary analyses were conducted using the MEGA11 (Version 11) software [42]. The 30 protein-coding nucleotide sequences for orthologous *dd4*-like and *tth*-like genes from 16 Diptera species (Figure S2) were aligned using the MUSCLE tool [43], provided by MEGA11. Only ORFs were included in the analysis. All the positions containing gaps and missing data were eliminated (complete deletion option). There were a total of 726 positions in the final dataset. Based on the alignment of sequences using the estimation of bioinformatic criteria, an optimal Kimura 2-parameter model [44] for calculating pairwise distances between sequences was selected. The evolutionary history was inferred by using the Maximum Likelihood method. The bootstrap test, conducted with 1000 replicates, was employed to provide support for the Maximum Likelihood phylogenetic tree. The tree was drawn to scale, with branch lengths measured in the number of substitutions per site (Figure S2A).

#### 4.1.5. Analysis of Synteny

The DiGAlign software version 2.0 (https://www.genome.jp/digalign/, accessed on 6 November 2024) [45] was used to analyze synteny between genomic fragments from four mosquito species: *Culex pipiens pallens*, *Culex quinquefasciatus*, *Aedes aegypti*, and *Aedes albopictus*. Genomic sequences of 200 kb, including *tth*-like loci, were retrieved from the NCBI database and subjected to analysis. The tBLASTx algorithm was selected as the most appropriate.

### 4.2. Plutella xylostella Transcripts Validation

Source of *Plutella xylostella* material: Diamondback moth (*Plutella xylostella*) larvae and images were collected from experimental cabbage fields of the Federal Scientific Vegetable Center (FSBSI FSVC), Moscow Region, Russia, and frozen in liquid nitrogen.

#### RNA Extraction, cDNA Synthesis, and Reverse Transcription-PCR

Total RNA was extracted with RNAzol^®^ RT (MRC, Cincinnati, OH, USA) according to the manufacturer’s instructions. Samples with a 260/280 ratio between 1.8 and 2.2 were used for the subsequent analyses. RNA integrity was assessed by using 1.2% agarose gel electrophoresis, staining with ethidium bromide, and visualization with UV light. cDNA was synthesized from 2 µg of total RNA using an MMLV-RT kit with random priming (Evrogen, Moscow, Russia). The cDNA was diluted five-fold and subsequently used in reverse transcription-PCR using BioMaster LR HS-PCR (2×) mix (Biolabmix, Novosibirsk, Russia) with the addition of 10%DMSO. The primers were as follows: PlxFor1 5′-cgacacatcgtcctccgact, PlxFor6 5′-atgcagtcttgcgttgcgtct, PlxRev4 5′-ttcactagcgcttgctggtat, and PlxRev7 5′-accaatccgtggtgcgagaa (Figure S5A). The fragment resulting from PCR with the PlxFor1 and PlxRev7 primers was subcloned into pGEM-T easy vector (Promega, Madison, WI, USA), sequenced, and submitted to GenBank under the accession number OQ559524.

## 5. Conclusions

The analysis of the domain organization of protein products from the *d4* gene family across the evolutionary lineages, from non-bilaterians to vertebrates, has led to the following conclusions: (1) In all evolutionary lineages, single-copy *d4* genes retain the most complete exon composition encoding the characteristic domains of full-length D4 family proteins. However, the domain organization of the protein products of these genes can vary across splice isoforms. The absence of the Kruppel-type ZF domain is widespread across various lineages starting with Cnidaria. In some species, such as the nematode *C. elegans* and certain insects, isoforms lacking the D4 domain have emerged. (2) During evolution, gene duplications occurred in arthropods and vertebrates, followed by the divergence of paralogs based on their ability to generate specific splice isoforms, particularly those lacking the D4 domain. However, it has been observed that in certain species of insects (Diptera) and arachnids (spider mite, *T. urticae*), as well as in some fish (*O. melastigma*), one of the gene copies has undergone a loss of exons encoding the D4 domain. Our findings indicate that in fish, such genes directly inherit the structure of the DPF3a isoform from the ancestral paralog, suggesting that all paralogs lacking exons encoding the D4 domain may similarly inherit the structure and function of the corresponding isoforms. (3) The order Diptera is unique among insects in that it contains two *d4*-related paralogs, *tth*-like and *dd4*-like, while other insects possess a single-copy gene that can generate isoforms lacking the D4 domain, including DPF3a-like isoforms. We propose that the Drosophila *tth* paralog may assume the function of the DPF3a-like isoform, originating from the ancestral single-copy gene. (4) The broad distribution of DPF3a isoforms in vertebrates and similarly organized DPF3a-like isoforms in arthropods suggests that these isoforms are functionally significant and likely serve similar functions across different animal groups. However, sequence divergence in the C-termini among DPF3a-like isoforms in insects indicates potential functional diversity. Therefore, the primary function of these isoforms appears to be associated with their N-terminal regions, specifically the 2/3 domain. (5) Unlike the loss of exons encoding the D4 domain or the Kruppel-type zinc finger observed in some *d4* gene paralogs, no loss of exons encoding the 2/3 domain has been detected. This leads us to conclude that the 2/3 domain is the key defining characteristic of the *d4* gene family. (6) Based on the ortholog conjecture, the divergence between the amino acid sequences of the 2/3 domains of the paralogous D4-related proteins in dipterans, crustaceans, and vertebrates has functional significance. This points to a potential neofunctionalization of the domains, which may be related to their specialization in interacting with distinct proteins, such as with the homologs of NF-κB transcription factors.

## Figures and Tables

**Table 1:** *d4*-related genes in the genomes of the selected species according to the NCBI database *.

Taxonomy	Species	Gene Copy Number
**Non-bilaterian**		
Placozoa	*Trichoplax adhaerens*	1
Porifera	*Amphimedon queenslandica*	1
Cnidaria	*Hydra vulgaris*	1
**Protostomes**		
Brachiopoda	*Lingula anatina*	1
Mollusca	*Pomacea canaliculata*	1
Nematoda	*Caenorhabditis elegans*	1
Crustacea	*Daphnia magna*	1
	*Eurytemora affinis*	2
	*Homarus americanus*	2
Chelicerata	*Ixodes scapularis*	1
	*Centruroides sculpturatus*	2
	*Limulus polyphemus*	3
	*Panonychus citri*	3
	*Tetranychus urticae*	4
Insects (Hemimetabola)	*Zootermopsis nevadensis*	1
	*Frankliniella occidentalis*	1
Insects (Holometabola)	*Eufriesea mexicana*	1
	*Onthophagus taurus*	1
	*Plutella xylostella*	1
	*Anopheles gambiae*	1
	*Drosophila melanogaster*	2
**Deuterostomes**		
Echinodermata	*Asterias rubens*	1
Hemichordata	*Saccoglossus kowalevskii*	1
Chordata (Invertebrata)	*Branchiostoma floridae*	1
	*Ciona intestinalis*	1
Chordata (Vertebrata)	*Petromyzon marinus*	2
	*Carcharodon carcharias*	3
	*Danio rerio*	4
	*Rana temporaria*	3
	*Lacerta agilis*	3
	*Gallus gallus*	3
	*Homo sapiens*	3

*—the full list of species and their nuclear genomes analyzed in this work is available in Table S1 in the Supplementary Materials.

**Table 2:** Domain organization of proteins/isoforms encoded by *d4*-related genes in invertebrates.

Order	Species	Gene Symbol	Proteins/Isoforms	Protein a.a.	Domains		
					2/3	C2H2	D4
**Non-bilateria**							
Cnidaria	*Nematostella vectensis*	*LOC5513005*	XP_032238404.1	407	+	+	+
			XP_032238405.1	360	+	-	+
**Bilateria/Protostomia**							
Mollusca	*Biomphalaria glabrata*	*LOC106077827*	XP_055877054.1	381	+	+	+
			XP_013094154.1	371	+	-	+
Nematoda	*Caenorhabditis elegans*	*dpff-1*	NP_498281.2	372	+	+	+
			NP_001359900.1	192	+	-	-
**Bilateria/Protostomia/Insects**							
Lepidoptera	*Plutella xylostella*	*LOC105388869*	XP_037965301.2	503	+	+	+
			WDY85960.1	505	+	+	C2C2
Hymenoptera	*Eufriesea mexicana*	*LOC108553237*	XP_017763548.1	527	+	+	+
			XP_017763549.1	476	+	-	+
			OAD52898.1	396	+	+	C2C2
Coleoptera	*Onthophagus taurus*	*LOC111416346*	XP_022904108.1	516	+	+	+
			XP_022904139.1	414	+	-	+
			XP_022904123.1	457	+	+	C2C2
	*Tribolium castaneum*	*LOC655725*	XP_008200071.1	525	+	+	+
			KYB28897.1	472	+	+	C2C2
Thysanoptera	*Frankliniella occidentalis*	*LOC113203741*	XP_026274369.1	533	+	+	+
			XP_026274377.1	401	+	-	+
			XP_052124846.1	411	+	+	-
Blattodea	*Zootermopsis nevadensis*	*LOC110835538*	XP_021931530.1	544	+	+	+
			XP_021931534.1	447	+	-	+
			XP_021931533.1	477	+	+	C2C2
Diptera	*Drosophila melanogaster*	*toothrin*	NP_001285216.1	428	+	-	-
		*drosophila d4*	NP_610163.1	497	+	-	+
			NP_724405.2	339	-	-	+
	*Drosophila* *suzuki*	*LOC108013735*	XP_016935173.1	434	+	-	-
		*LOC108007830*	XP_016927082.1	496	+	-	+
			XP_036677958.1	339	-	-	+
	*Drosophila eugracilis*	*LOC108118381*	XP_017086545.1	429	+	-	-
		*LOC108104156*	XP_017065546.1	494	+	-	+
			XP_017065547.1	391	-	-	+
	*Aedes aegypti*	*LOC5573515*	XP_001660913.1	317	+	-	-
		*LOC5574646*	XP_001661565.1	433	+	-	+
	*Anopheles gambiae*	*LOC1274933*	XP_061497220.1	529	+	-	+
**Bilateria/Protostomia/Arachnida**							
Acari	*Tetranychus urticae*	*LOC107371702*	XP_015795331.1	374	+	+	-
		*LOC107359508*	XP_015781480.1	361	+	-	+
		*LOC107366194*	XP_015789261.1	528	+	+	+
		*LOC107367175*	XP_015790346.1	416	+	+	+
	*Panonychus citri*	*LOC128397756*	XP_053214481.1	389	+	-	+
		*LOC128395291*	XP_053211672.1	534	+	+	+
		*LOC128395295*	XP_053211679.1	484	+	+	+
**Bilateria/Protostomia/Crustacea**							
Branchiopoda	*Daphnia magna*	*LOC116928451*	XP_032791427.2	500	+	+	+
			XP_045033736.1	426	+	-	+
Copepoda	*Eurytemora affinis*	*LOC111700562*	XP_023327298.1	448	+	+	+
		*LOC111695774*	XP_023320978.1	394	+	-	+
Decapoda	*Homarus americanus*	*LOC121855616*	XP_042206607.1	527	+	+	+
			XP_042206610.1	464	+	+	C2C2
			XP_042206611.1	441	+	-	+
		*LOC121878412*	XP_042240571.1	431	+	+	+
			XP_042240572.1	391	+	-	+
	*Penaeus japonicus*	*LOC122250696*	XP_042868189.1	464	+	+	+
			XP_042868193.1	376	+	-	+
		*LOC122250070*	XP_042867286.1	517	+	+	+
			XP_042867289.1	431	+	-	+
			XP_042867288.1	443	+	+	C2C2
	*Portunus trituberculatus*	*LOC123516819*	XP_045132453.1	495	+	+	+
			XP_045132459.1	426	+	-	+
		*LOC123508560*	XP_045118251.1	542	+	+	+
			XP_045118259.1	468	+	+	C2C2
Amphipoda	*Hyalella azteca*	*LOC108677705*	XP_047740582	469	+	+	+
		*LOC108672214*	XP_018015338.1	550	+	+	+
**Bilateria/Deuterostomia**							
Echinodermata	*Asterias rubens*	*LOC117289848*	XP_033626936.1	424	+	+	+
			XP_033626944.1	383	+	-	+

Designations: 2/3—N-terminal 2/3 domain; C2H2—Kruppel-type zinc finger; D4—C-terminal D4 domain; C2C2—a remnant of the D4 domain (1/2 PHD1) that contains four zinc-binding Cys residues, a hallmark for DPF3a-like isoforms. The presence or absence of the domain is indicated by “+” or “-”.

**Table 3:** Domain composition of isoforms encoded by *d4*-related paralogs in Chordata/Vertebrata.

Order	Species	Gene Symbol	Isoforms	Protein a.a.	Domains		
					2/3	C2H2	D4
Hyperoartia	*Petromyzon marinus*	*LOC116939432*	XP_032803643.1	418	+	+	+
			XP_032803641.1	432	+	Ins	+
			XP_032803650.1	334	+	+	C2C2
			XP_032803645.1	392	+	Ins	C2C2
		*LOC116954017*	XP_032830294.1	408	+	+	+
Actinopterygii	*Danio rerio*	*dpf1*	NP_001314998.1	407	+	+	+
			NP_001025375.2	302	+	+	-
		*dpf2*	NP_001007153.1	400	+	+	+
			XP_005165359.1	414	+	Ins	+
			XP_021331801.1	324	TR	Ins	+
		*dpf2l*	NP_997861.2	405	+	+	+
		*dpf3*	XP_005160800.1	377	+	+	+
			NP_001104639.1	391	+	Ins	+
			XP_009293039.1	358	+	Ins	C2C2
			XP_005160801.1	344	+	+	C2C2
	*Oryzias melastigma*	*dpf1*	XP_024132703.1	406	+	+	+
		*dpf2*	XP_024119682.1	400	+	+	+
			XP_024119664.1	414	+	Ins	+
		*dpf2l*	XP_024139692.1	408	+	+	+
			XP_024139696.1	362	+	-	+
		*dpf3* *	XP_024114574.1	362	+	+	C2C2
			XP_024114494.1	376	+	Ins	C2C2
Amphibia	*Rana temporaria*	*dpf1*	XP_040179057.1	410	+	+	+
			XP_040179060.1	352	TR	+	+
		*dpf2*	XP_040184580.1	388	+	+	+
			XP_040184579.1	402	+	Ins	+
		*dpf3*	XP_040189603.1	396	+	Ins	+
			XP_040189604.1	382	+	+	+
			XP_040189606.1	344	+	+	C2C2
			XP_040189605.1	358	+	Ins	C2C2
Reptiles	*Lacerta agilis*	*dpf1*	XP_033014410.1	366	+	+	+
			XP_033014409.1	330	TR	+	+
		*dpf2*	XP_032992316.1	391	+	+	+
			XP_032992315.1	405	+	Ins	+
		*dpf3*	XP_033002348.1	429	+	+	+
			XP_033002385.1	322	TR	+	+
			XP_033002368.1	378	TR	+	+
			XP_033002357.1	408	+	+	C2C2
Aves	*Gallus gallus*	*dpf1*	NP_989971.1	380	+	+	+
		*dpf2*	NP_989662.1	405	+	Ins	+
		*dpf3*	NP_989970.2	427	+	+	+
			XP_040556501.1	371	TR	+	+
			XP_040556497.1	391	+	+	C2C2
Mammalia	*Mus musculus*	*dpf1*	NP_001390141.1	341	+	+	+
			NP_001390147.1	285	TR	+	+
		*dpf2*	NP_035392.1	391	+	+	+
			NP_001278007.1	405	+	Ins	+
		*dpf3*	NP_001254554.1	378	+	+	+
			NP_478119.1	356	+	+	C2C2
	*Homo sapiens*	*dpf1*	XP_006723470.1	398	+	+	+
			XP_006723473.1	242	-	+	+
			NP_001128628.1	332	TR	+	+
		*dpf2*	NP_006259.1	391	+	+	+
			NP_001317237	405	+	Ins	+
		*dpf3*	NP_001267471	378	+	+	+
			NP_036206	357	+	+	C2C2

Designations: 2/3—N-terminal 2/3 domain; C2H2—Sfp1/Kruppel-type zinc finger; D4—C-terminal D4 domain. The presence or absence of the domain is indicated by “+” or “-” respectively. TR—N-terminally truncated 2/3 domain (NLS is retained). Ins—an insertion between the pair of Cys residues in C2H2 of Sfp1/Kruppel–like zinc finger. C2C2—a remnant of the D4 domain that contains four zinc-binding Cys residues (1/2 PHD1) and XZ stretch of amino acids (a hallmark for DPF3a-like isoforms). The asterisk “*” marks the gene encoding only the DPF3a protein isoform.

## Data Availability

Data is contained within the article and Supplementary Materials.

## Supplementary Materials

The following supporting information can be downloaded at: https://www.mdpi.com/article/10.3390/ijms252413394/s1.
