# Supplementary material for: The Drosophila toothrin Gene Related to the d4 Family Genes: An Evolutionary View on Origin and Function
Source: Int J Mol Sci. 2024 Dec 13;25(24):13394. doi: 10.3390/ijms252413394 (PMC11678306; doi:10.3390/ijms252413394)
Supplement: Supplementary file 1 [file ijms-25-13394-s001.zip › Figure S1.pdf]

(A)

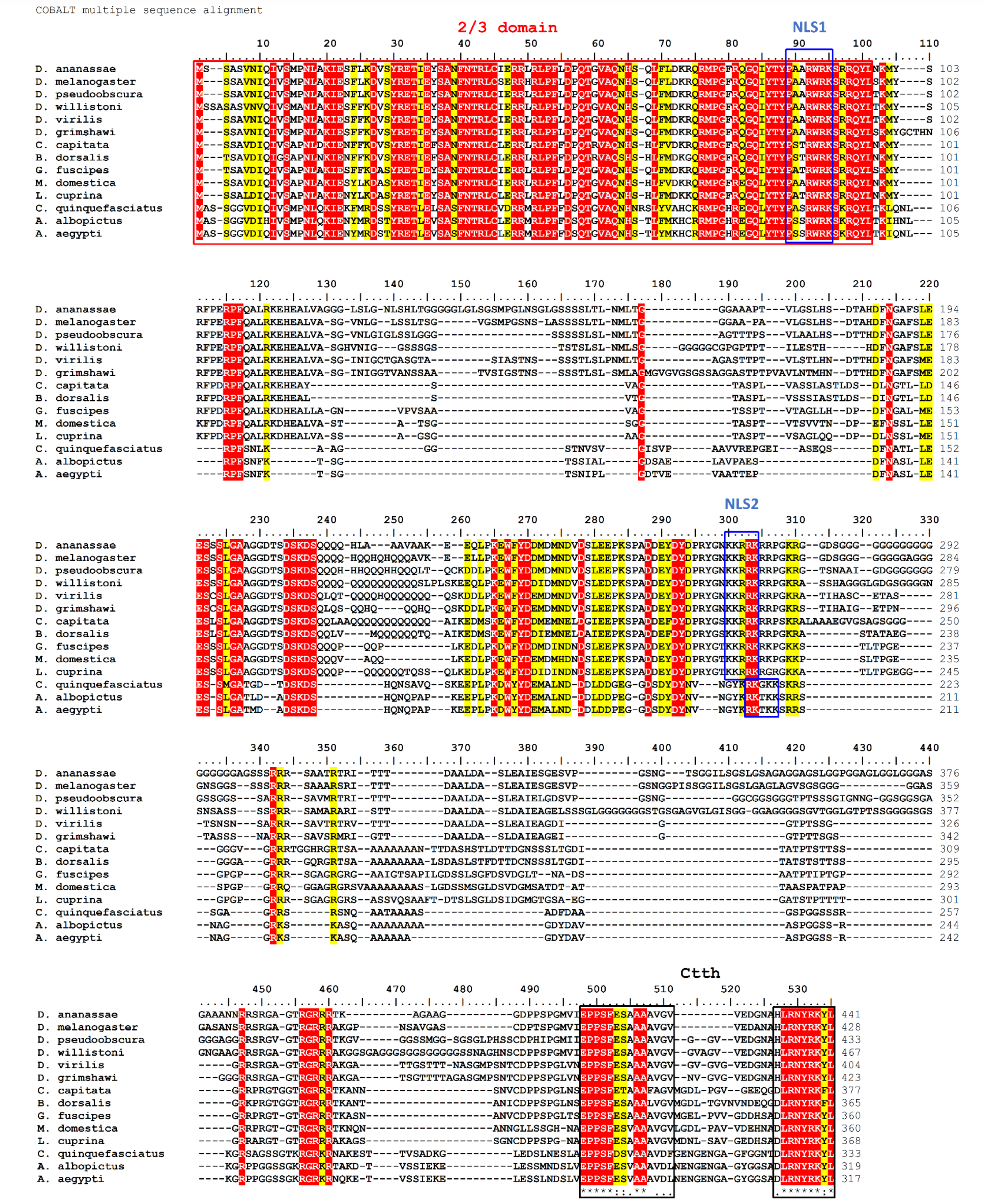
COBALT multiple sequence alignment
2/3 domain
NLS1
NLS2
Ctth
D. ananassae
D. melanogaster
D. pseudoobscura
D. willistoni
D. virilis
D. grimshawi
C. capitata
B. dorsalis
G. fuscipes
M. domestica
L. cuprina
C. quinquefasciatus
A. albopictus
A. aegypti

(B)

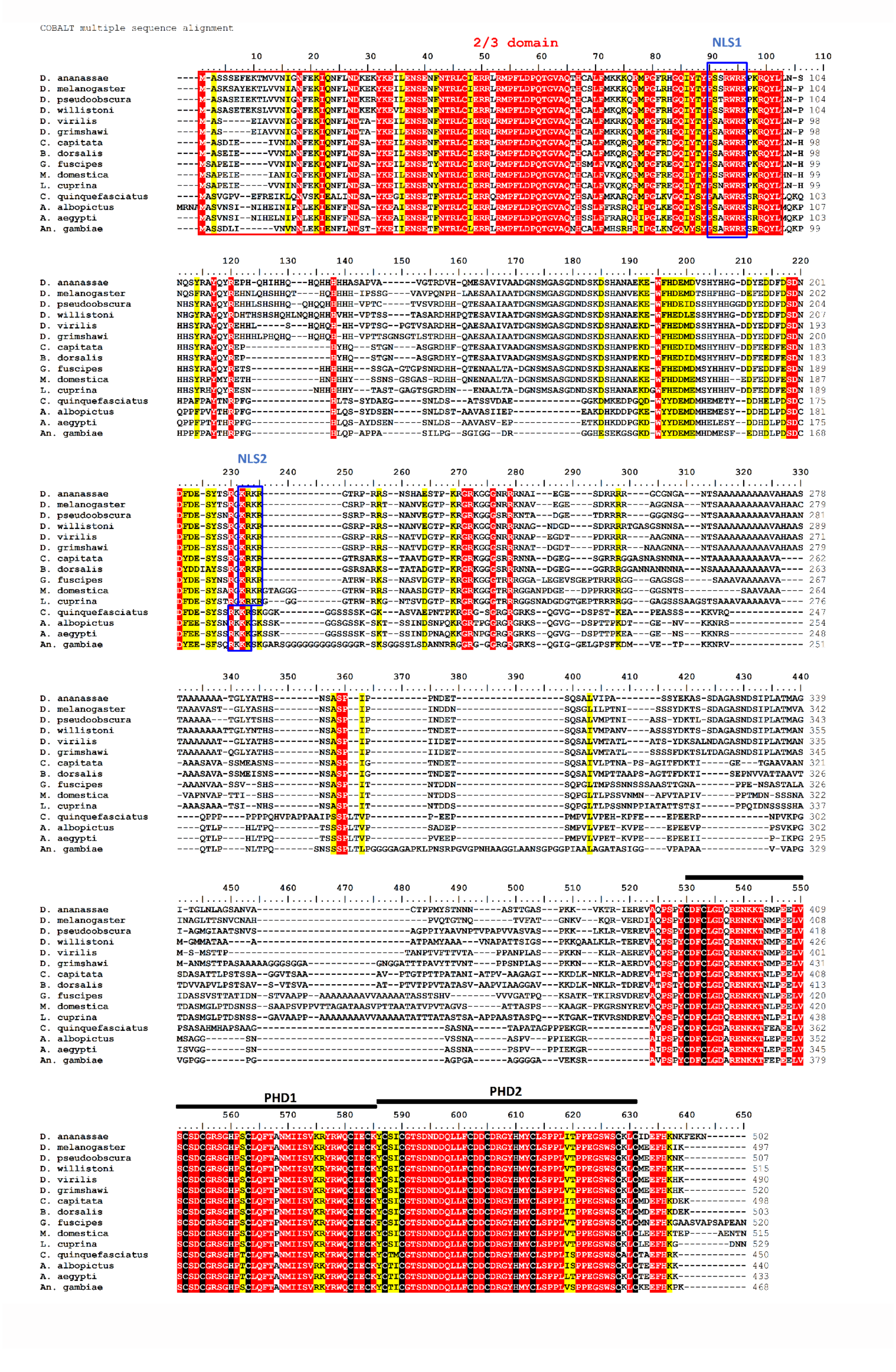
COBALT multiple sequence alignment
2/3 domain
NLS1
NLS2
PHD1
PHD2
D. ananassae
D. melanogaster
D. pseudoobscura
D. willistoni
D. virilis
D. grimshawi
C. capitata
B. dorsalis
G. fuscipes
M. domestica
L. cuprina
C. quinquefasciatus
A. albopictus
A. aegypti
An. gambiae

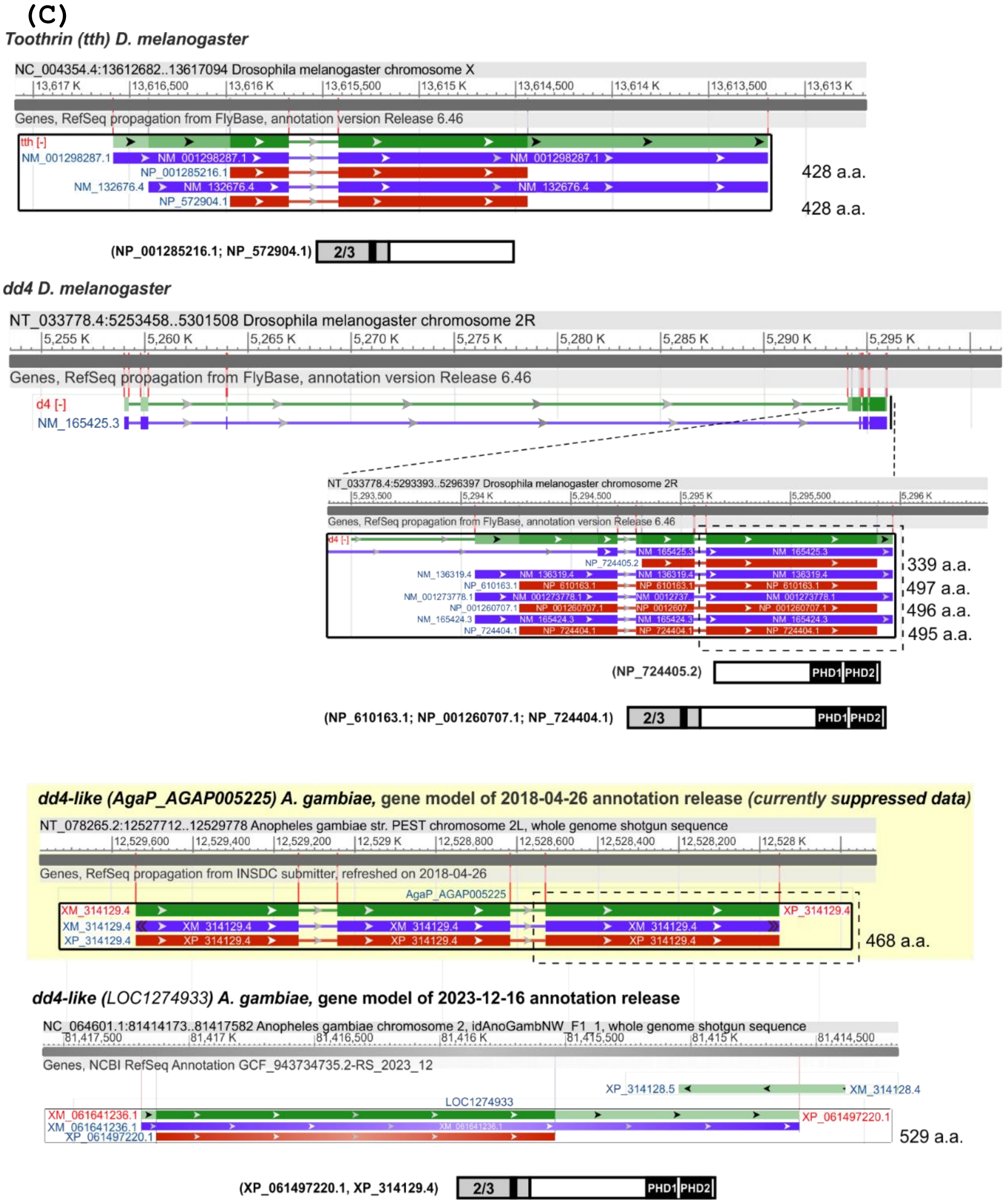

Figure S1. (A, B) Conservation of the amino acid sequences of TTH-like and DD4-like proteins in Diptera species. (C) The organization of *dd4* and *tth* genes in *Drosophila melanogaster* and *dd4*-like gene of *Anopheles gambiae*.

Multiple sequence alignment of dipteran TTH-like **(A)** and DD4-like **(B)** proteins. The conserved regions highlighted in gray: (*) – conserved residue, (:) – scoring > 0.5, (.) – scoring ≤ 0.5). NLS1 and NLS2 are blue-framed. Two conserved C-terminal motifs unique to dipteran TTH-like proteins are in black frames. Species are as follows: *D. ananassae* **(***Drosophila ananassae), D. mel***. (***Drosophila melanogaster), D. pseud. (Drosophila pseudoobscura), D. willistoni (Drosophila willistoni), D. virilis (Drosophila virilis), D. grimshawi (Drosophila grimshawi), C. capitata (Ceratitis capitata), B. dorsalis (Bactrocera dorsalis), G. fuscipes (Glossina fuscipes), M. domestica (Musca domestica), L. cuprina (Lucilia cuprina), C. quinq. (Culex quinquefasciatus), A. albopictus (Aedes albopictus), A. aegypti (Aedes aegypti).*

(C) Organization of the *tth* and *dd4* genes of *Drosophila melanogaster* and *dd4*-like gene *of Anopheles gambiae*. The gene models are displayed according to the NCBI: Genome Regions, Transcripts and Products Viewer: the exon-intron structure of the genes is highlighted in green, the protein products are red-colored.

In the yellow field is shown currently suppressed in NCBI database the *Anopheles gambiae* dd4-like gene model which predicts the three-exonic transcript XM_314129.4 (corresponding TSA transcript is GIBN01005664.1).

Below is shown the NCBI gene model *LOC1274933* of the *dd4*-like gene of *Anopheles gambiae*, which predicts the intronless transcript XM_061641236.1 (corresponding TSA transcript is GKOR01001022.1).

A schematic representation of the domain organization of the protein products of the genes is shown below the gene models. The 2/3 domain caring NLS (black bar) is filled with gray, the PHD fingers (PHD1, PHD2) are filled with black.
