# Supplementary material for: The Drosophila toothrin Gene Related to the d4 Family Genes: An Evolutionary View on Origin and Function
Source: Int J Mol Sci. 2024 Dec 13;25(24):13394. doi: 10.3390/ijms252413394 (PMC11678306; doi:10.3390/ijms252413394)
Supplement: Supplementary file 1 [file ijms-25-13394-s001.zip › Figure S2.pdf]

(A)

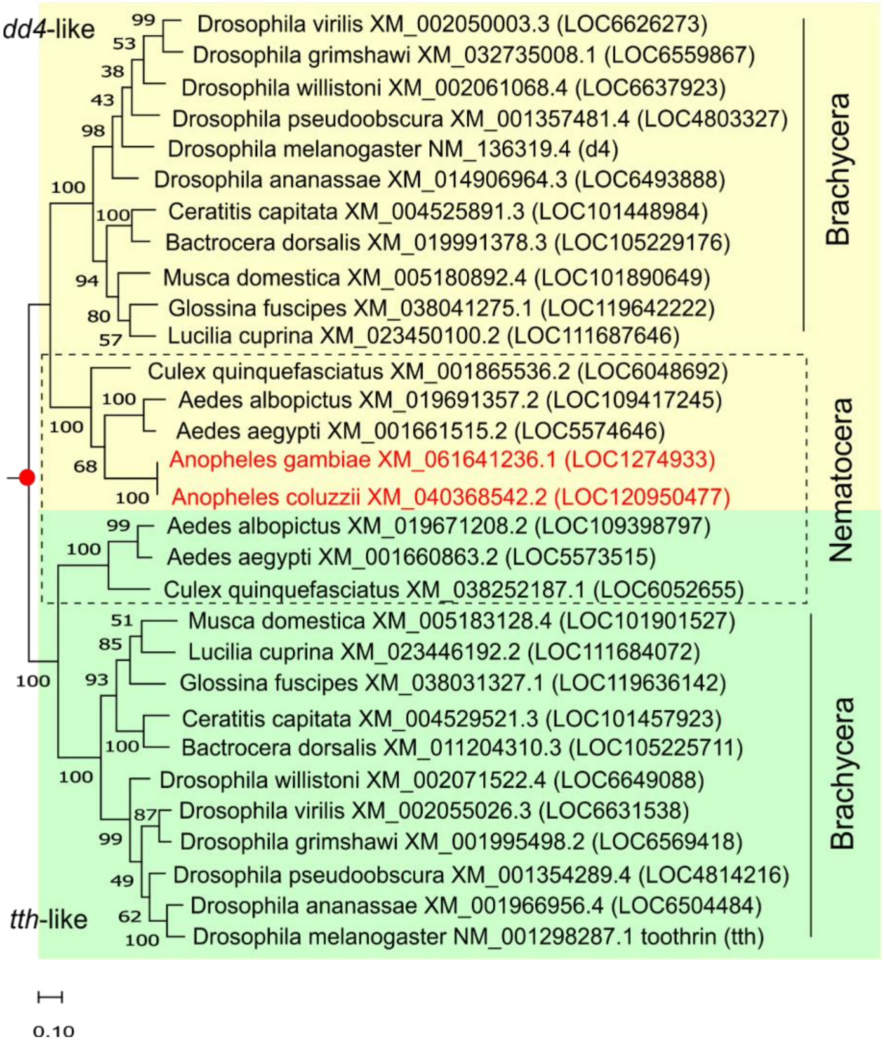

Figure S2A. Maximum Likelihood tree showing the relationship between the transcripts of the *dd4*-like and *tth*- like orthologs in Diptera.

The red circle indicates the duplication point. Transcripts of tth-like genes and dd4-like genes are shown in green and yellow fields, respectively. The dashed frame indicates the transcripts of the d4 family genes of the mosquito clades. Species names with a single dd4-like gene are colored in red. The percentage of replicate trees in which the associated taxa clustered together in the bootstrap test (1,000 replicates) are shown next to the branches. The scale bar indicates the number of substitutions per site.

The species names and mRNA/Gene NCBI/Genebank accession numbers are as follows:

***dd4*-like:** *Drosophila ananassae* (XM_014906964.3, *LOC6493888*), *Drosophila melanogaster* (NM_136319.4, *d4*); *Drosophila pseudoobscura* (XM_001357481.4, *LOC4803327*); *Drosophila willistoni* (XM_002061068.4, *LOC6637923*); *Drosophila grimshawi* (XM_032735008.1, *LOC6559867*); *Drosophila virilis* (XM_002050003.3, *LOC6626273*); *Glossina fuscipes* (XM_038041275.1, *LOC119642222*); *Musca domestica* (XM_005180892.4, *LOC101890649*); *Lucilia cuprina* (XM_023450100.2, *LOC111687646*); *Ceratitis capitata* (XM_004525891.3, *LOC101448984*); *Bactrocera dorsalis* (XM_019991378.3, *LOC105229176*); *Culex quinquefasciatus* (XM_001865536.2, *LOC6048692*); *Aedes albopictus* (XM_019691357.2, *LOC109417245*); *Aedes aegypti* (XM_001661515.2, *LOC5574646*); *Anopheles gambiae* (XM_061641236.1, *LOC1274933*); *Anopheles coluzzii* (XM_040368542.2, *LOC120950477*).

***tth*-like:** *Drosophila melanogaster* (NM_001298287.1, *tth*); *Drosophila ananassae* (XM_001966956.4, *LOC6504484*); *Drosophila pseudoobscura* (XM_001354289.4, *LOC4814216*); *Drosophila willistoni* (XM_002071522.4, *LOC6649088*); *Drosophila grimshawi* (XM_001995498.2, *LOC6569418*); *Drosophila virilis* (XM_002055026.3, *LOC6631538*); *Glossina fuscipes* (XM_038031327.1, *LOC119636142*); *Lucilia cuprina* (XM_023446192.2, *LOC111684072*); *Musca domestica* (XM_005183128.4, *LOC101901527*); *Bactrocera dorsalis* (XM_011204310.3, *LOC105225711*); *Ceratitis capitata* (XM_004529521.3, *LOC101457923*); *Culex quinquefasciatus* (XM_038252187.1, *LOC6052655*); *Aedes aegypti* (XM_001660863.2, *LOC5573515*); *Aedes albopictus* (XM_019671208.2, *LOC109398797*).

(B)

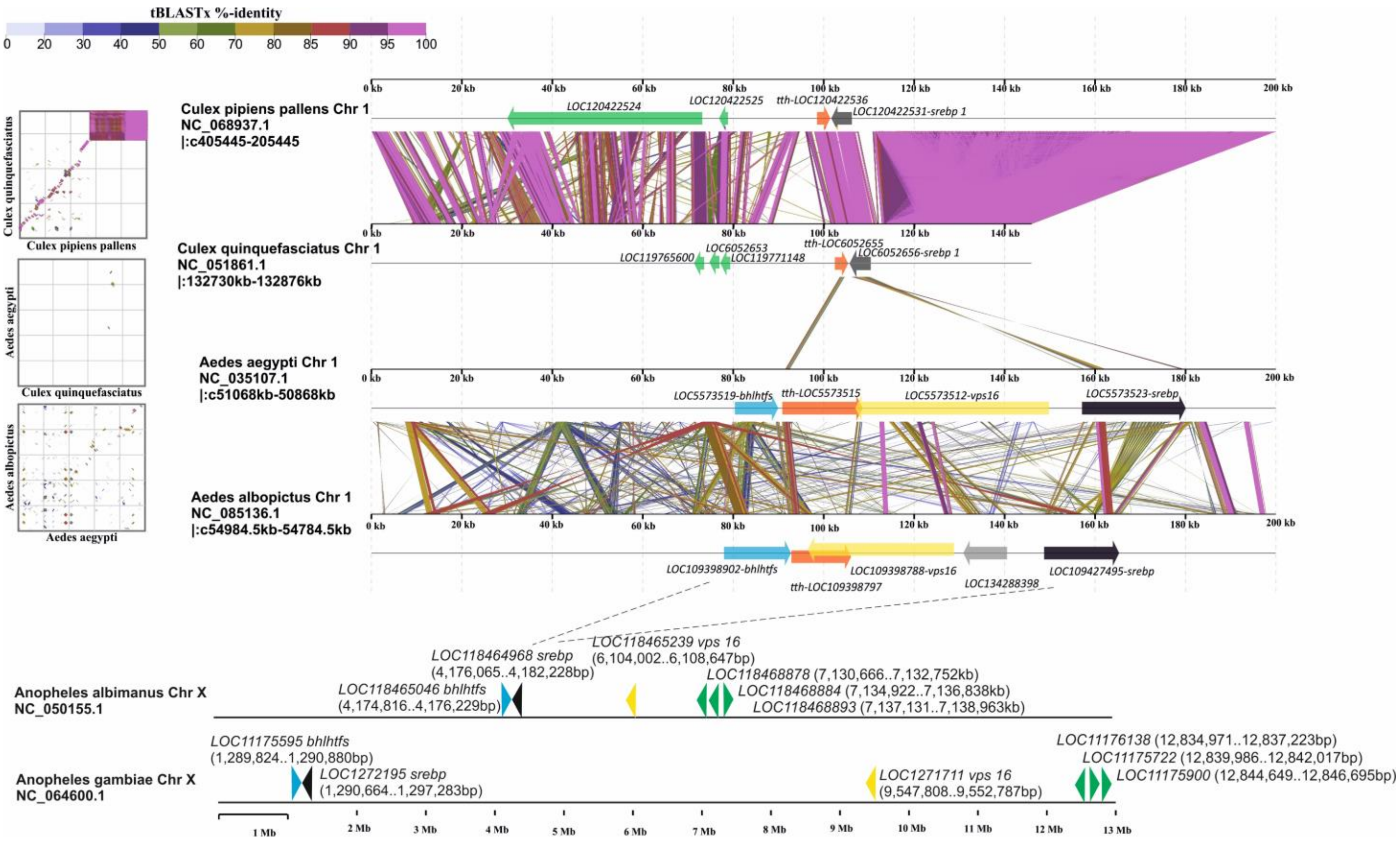

Figure S2B. Scenarios of *tth*-like gene loss in *Anopheles*.

Ruler scales show every 20 kb in *Aedes* and *Culex* genomic fragments and every 1 Mb in *Anopheles* genomic fragments. The DNA sequences are represented by horizontal black lines. Genes are depicted by arrows: red - *tth*-like , yellow - *vpsp 16* (*vacuolar protein sorting-associated protein 16 homolog*), blue - *bhlhtfs* (*basic helix-loop-helix transcription factor scleraxis-like*), black - *srebp* (*sterol regulatory element-binding protein 1-like*), green – homologous *tth* anchor genes of *Culex*. Related genes are filled in same colors. The gene with no counterparts is highlighted in gray. The 200 kb nucleotide sequences possessing the *tth*-like gene approximately in the center were retrieved from the *Aedes aegypti*, *Aedes albopictus*, and *Culex pipens pallens* genomes provided by NCBI. It should be noted that only 146 kb sequence was obtained from the *Culex quinquefasciatus* genome. Dot plots and synteny maps show the relationship of syntenic regions between sequences. The black dashed lines show orthologs rearrangement.
