# Supplementary material for: The Drosophila toothrin Gene Related to the d4 Family Genes: An Evolutionary View on Origin and Function
Source: Int J Mol Sci. 2024 Dec 13;25(24):13394. doi: 10.3390/ijms252413394 (PMC11678306; doi:10.3390/ijms252413394)
Supplement: Supplementary file 1 [file ijms-25-13394-s001.zip › Figure S3.pdf]

NLS's inside 2/3 domains

```
M.musculus DPF1   NP_001390141.1  72-YTYPARCWRKKRRLNI-87
M.musculus DPF2   NP_035392.1     72-YSYPARRWRKKRRAHP-87
M.musculus DPF3   NP_001254554.1  72-YTYPARCWRKKRRLHP-87
E.mexicana        XP_017763548.1  89-YTYPSKRWRKKRRQYL-104
O.taurus          XP_022904123.1  80-YTYPRQRWRKKRRQYL-96
P.xylostella      XP_037965301.2  90-YSYPSQRWRKARRQYL-105
D.mel D4          NP_610163.1     86-YTYPSSRWRKPKRQYL-101
D.mel TTH         NP_001285216.1  82-YTYPAARWRKSRRQYL-97
                                       *:**    ***  :*
```

>NP_001390141.1 Neuro-d4/DPF1
[Mus musculus]
NUCDISC:
pat4: RKKR (5) at 80
pat4: KKRR (5) at 81
pat4: KPKK (4) at 213
pat7: none
bipartite: none
content of basic residues: 13.8%
NLS Score: 0.40

>NP_035392.1 Ubi-d4/DPF2
[Mus musculus]
NUCDISC:
pat4: KRHR (3) at 59
pat4: RKKR (5) at 80
pat4: KKRR (5) at 81
pat4: PKRR (4) at 177
pat7: PARRWRK (4) at 75
pat7: PKRRGKG (5) at 177
bipartite: none
content of basic residues: 15.1%
NLS Score: 1.37

>NP_001254554.1 Cer-d4/DPF3
[Mus musculus]
NUCDISC:
pat4: KRHR (3) at 59
pat4: RKKR (5) at 80
pat4: KKRR (5) at 81
pat4: PKRK (4) at 166
pat4: RRRH (3) at 183
pat7: PKRKNRT (5) at 166
bipartite: none
content of basic residues: 14.3%
NLS Score: 1.21

>XP_017763548.1 PREDICTED:
zinc finger protein ubi-d4
[Eufriesea mexicana]
NUCDISC:
pat4: RKKR (5) at 97
pat4: KKRR (5) at 98
pat4: KRKR (5) at 181
pat4: RKRR (5) at 182
pat4: KRRK (5) at 183
pat4: RRKP (4) at 184
pat4: RKPR (4) at 185
pat7: PSKRWRK (4) at 92
pat7: PGGKRTK (3) at 205
bipartite:
KRRQYLMHYLHPKRGPR at 99
content of basic residues: 12.0%
NLS Score: 2.69

>XP_022904123.1 zinc finger protein
ubi-d4 A isoform X3
[Onthophagus taurus]
NUCDISC:
pat4: KRHR (3) at 68
pat4: RKRR (5) at 89
pat4: KRRR (5) at 90
pat4: KRRK (5) at 180
pat7: PRQRWRK (3) at 84
pat7: PYSKRTK (3) at 174
pat7: PGRRKGG (5) at 199
pat7: PGRRGRQ (4) at 336
bipartite: none
content of basic residues: 12.0%
NLS Score: 1.95

>XP_037965301.2 zinc finger protein
ubi-d4
[Plutella xylostella]
NUCDISC:
pat4: RRKK (5) at 191
pat4: RKKR (5) at 192
pat7: PSQRWRK (3) at 93
pat7: PKRGRAG (5) at 222
bipartite:
RKSAAPVDEGTPKRGRA at 211
content of basic residues: 12.3%
NLS Score: 1.34

>NP_610163.1 d4,
[Drosophila melanogaster]
NUCDISC:
pat4: RKPK (4) at 94
pat4: KPKR (4) at 95
pat4: KRKK (5) at 213
pat4: RPRR (4) at 219
pat4: RKRR (5) at 252
pat7: PSSRWRK (3) at 89
pat7: PKRGRKG (5) at 231
bipartite: RRTNANVEGTPKRGRKG at 221
bipartite: RRKNAVEGESDRKRRAG at 241
content of basic residues: 12.1%
NLS Score: 2.58

>NP_001285216.1 toothrin,
[Drosophila melanogaster]
NUCDISC:
pat4: RRHR (3) at 45
pat4: KKRR (5) at 258
pat4: KRRK (5) at 259
pat4: RRKR (5) at 260
pat4: RKRR (5) at 261
pat4: KRRP (4) at 262
pat7: PAARWRK (3) at 85
bipartite: none
content of basic residues: 11.9%
NLS Score: 1.48

Figure S3. Nuclear Localization Signals in D4 family proteins.
The conserved NLSs within the 2/3 domain of mouse DPF1-3 and Insect D4-related proteins are shown in the alignment at the top. The NLS motifs are highlighted in black. The species are as follows: *M. musculus (Mus musculus), E. mexicana (Eufriesea mexicana), O. Taurus (Onthophagus taurus), P. xyllostella (Plutella xylostella), D. mel (Drosophila melanodaster*).
Below, within the black frames, are the results of the query obtained from the Protein Subcellular Localization Prediction Tool PSORT II (https://www.genscript.com/tools/psort) [37]. The NLS motifs that have conserved positions within the 2/3 domain of the D4 family proteins are indicated in red. The NLS motifs that are not associated with the 2/3 domain and are conserved in insect proteins are indicated in blue.
