# Supplementary material for: The Drosophila toothrin Gene Related to the d4 Family Genes: An Evolutionary View on Origin and Function
Source: Int J Mol Sci. 2024 Dec 13;25(24):13394. doi: 10.3390/ijms252413394 (PMC11678306; doi:10.3390/ijms252413394)
Supplement: Supplementary file 1 [file ijms-25-13394-s001.zip › Figure S4.pdf]

Figure S4. The clustering of 2/3 domain sequences by paralogs.

The percent identity matrix of the aligned 2/3 domains, generated by the Clustal Omega program from UniProt resources [20]. Higher and lower percentages of identity are indicated by colors ranging from dark blue to light blue, respectively.

(A)

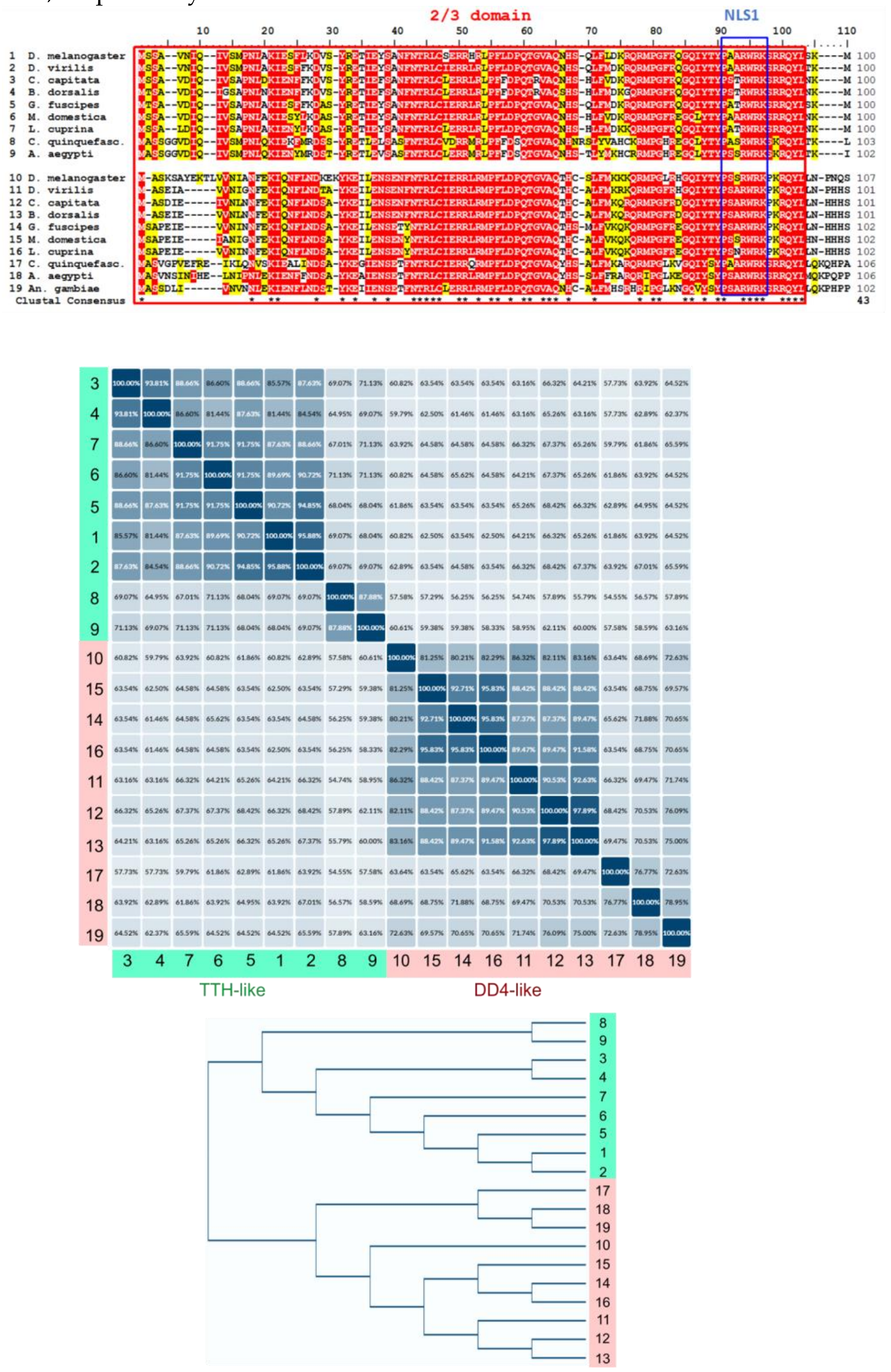

(A) alignment of 2/3 domains from TTH-like and DD4-like proteins of Diptera species: 1-9 TTH-like proteins: 1) *Drosophila melanogaster*, NP_001285216.1; 2) *Drosophila virilis*, XP_002055062.1; 3) *Ceratitis capitata*, XP_004529578.1; 4) *Bactrocera dorsalis*, XP_011202612.1; 5) *Glossina fuscipes*, XP_037887255.1; 6) *Musca domestica*, XP_005183185.1; 7) *Lucilia cuprina*, XP_023301960.1; 8) *Culex quinquefasciatus*, XP_038108115.1; 9) *Aedes aegypti*, XP_001660913.1; **10-19 DD4-like proteins:** 10) *Drosophila. melanogaster*, NP_610163.1; 11) *Drosophila virilis*, XP_002050039.1; 12) *Ceratitis capitata*, XP_004525948.1; 13) *Bactrocera dorsalis*, XP_011207592.1; 14) *Glossina fuscipes*, XP_037897203.1; 15) *Musca domestica*, XP_005180949.1; 16) *Lucilia cuprina*, XP_023305868.1; 17) *Culex quinquefasciatus*, XP_001865571.1; 18) *Aedes aegypti*, XP_001661565.1; 19) *Anopheles gambiae* XP_314129.4. The guide tree dendrogram at the bottom of the figure showing groups of closely related sequences.

(B)

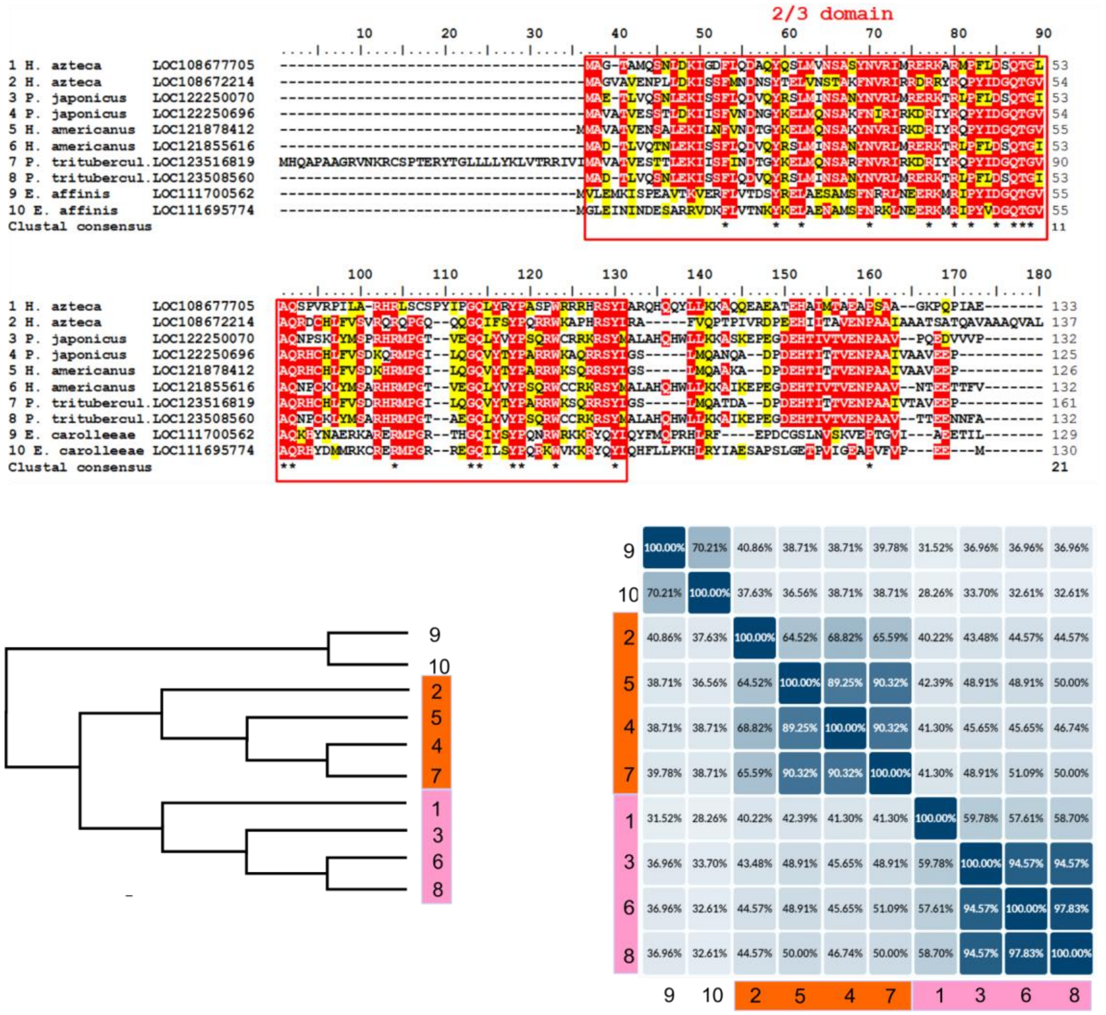

(B) alignment of 2/3 domains from crustacean paralogs.

Species and protein accession numbers are as follows: **1-10:** 1) *Hyalella Azteca*, XP_047740582.1; 2) *Hyalella Azteca*, XP_018015338.1; 3) *Panulirus japonicas*, XP_042867286.1; 4) *Panulirus japonicas*, XP_042868189.1; 5) *Homarus americanus*, XP_042240570.1; 6) *Homarus americanus*, XP_042206607.1; 7) *Portunus trituberculatus*, XP_045132453.1; 8) *Portunus trituberculatus*, XP_045118251.1; 9) *Eurytemora carolleeae*, XP_023327298.1; 10) *Eurytemora carolleeae*, XP_023320978.1. The guide tree dendrogram at the left of the matrix diagram showing groups of closely related sequences.

(C)

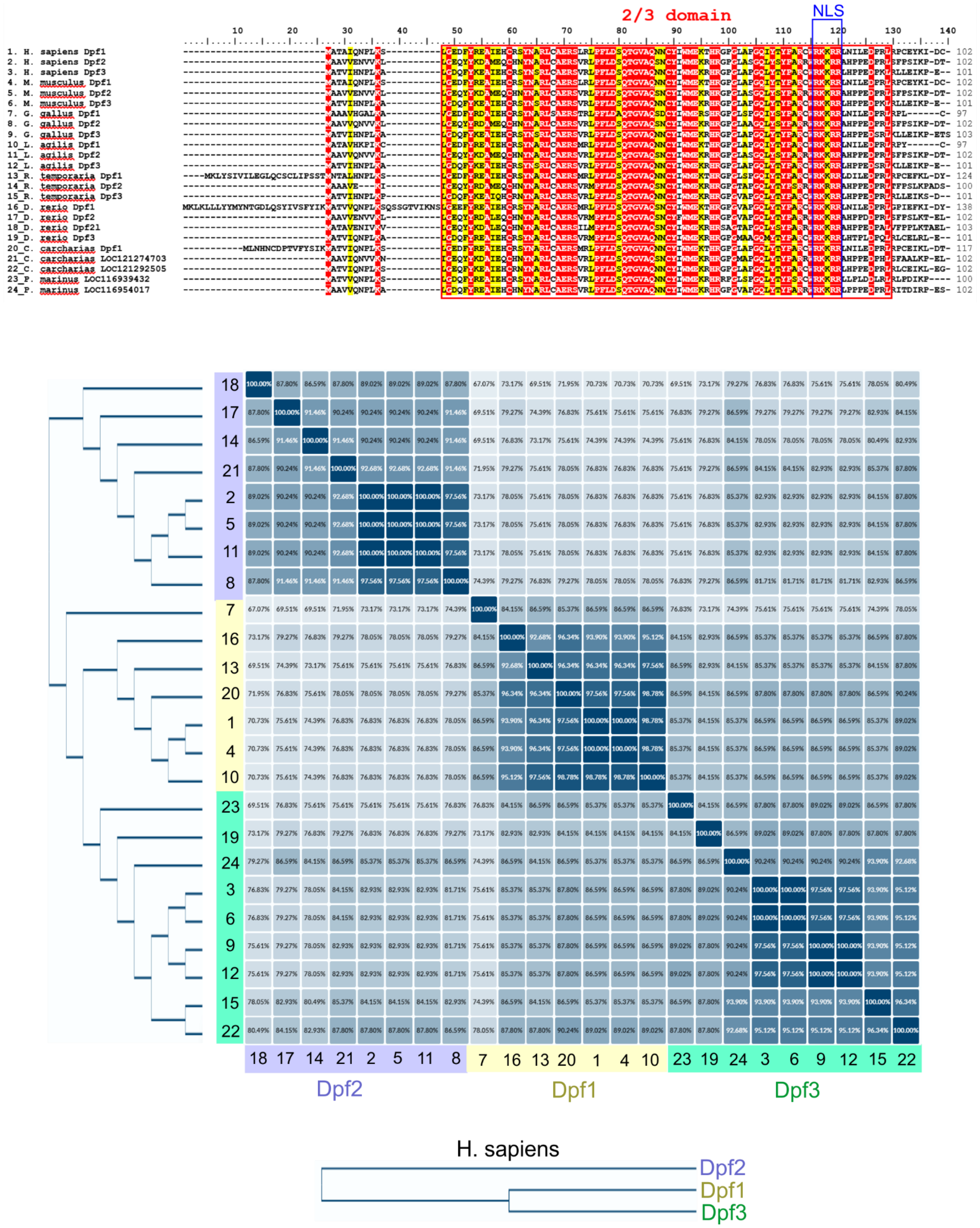

(C) alignment of 2/3 domains from vertebrata paralogs.

Species and protein accession numbers are as follows: **1-24**: 1) *Homo sapiens* DPF1 (XP_006723470.1),; 2) *Homo sapiens*, DPF2 (NP_006259.1); 3) *Homo sapiens* DPF3 (NP_001267471.1); 4) *Mus musculus* DPF1 (NP_001390141.1); 5) *Mus musculus* DPF2 (NP_035392.1); 6) *Mus musculus* (DPF3) NP_001254554.1; 7) *Gallus gallus* Dpf1 (NP_989971.1); 8) *Gallus gallus*, DPF2 (NP_989662.1); 9) *Gallus gallus* Dpf3 (NP_989970.2); 10) *Lacerta agilis* Dpf1 (XP_033014410.1); 11) *Lacerta agilis* Dpf2 (XP_032992316.1); 12 *Lacerta agilis* Dpf3 (XP_033002368.1); 13) *Rana temporaria* Dpf1 (XP_040179057.1); 14) *Rana temporaria*, Dpf2 (XP_040184580.1); 15) *Rana temporaria* Dpf3 (XP_040189604.1); 16) *Danio rerio* Dpf1 (NP_001314998.1); 17) *Danio rerio*, Dpf2 (NP_001007153.1); 18) *Danio rerio* Dpf2l (NP_997861.2); 19) *Danio rerio* Dpf3 (XP_005160800.1); 20) *Carcharodon carcharias* Dpf1 (XP_041035237.1); 21) *Carcharodon carcharias* ubi-d4-like (XP_041037955.1); 22) *Carcharodon carcharias* DPF3-like (XP_041070480.1); 23 *Petromyzon marinus* DPF3-like (XP_032830294.1); 24) *Petromyzon marinus* ubi-d4-like (XP_032803642.1). The guide tree dendrogram at the left of the matrix diagram showing groups of closely related sequences (at the bottom of the figure is the dendrogram showing relations between 2/3 domains of Homo sapiens paralogs).
