# Supplementary material for: The Drosophila toothrin Gene Related to the d4 Family Genes: An Evolutionary View on Origin and Function
Source: Int J Mol Sci. 2024 Dec 13;25(24):13394. doi: 10.3390/ijms252413394 (PMC11678306; doi:10.3390/ijms252413394)
Supplement: Supplementary file 1 [file ijms-25-13394-s001.zip › Figure S5.pdf]

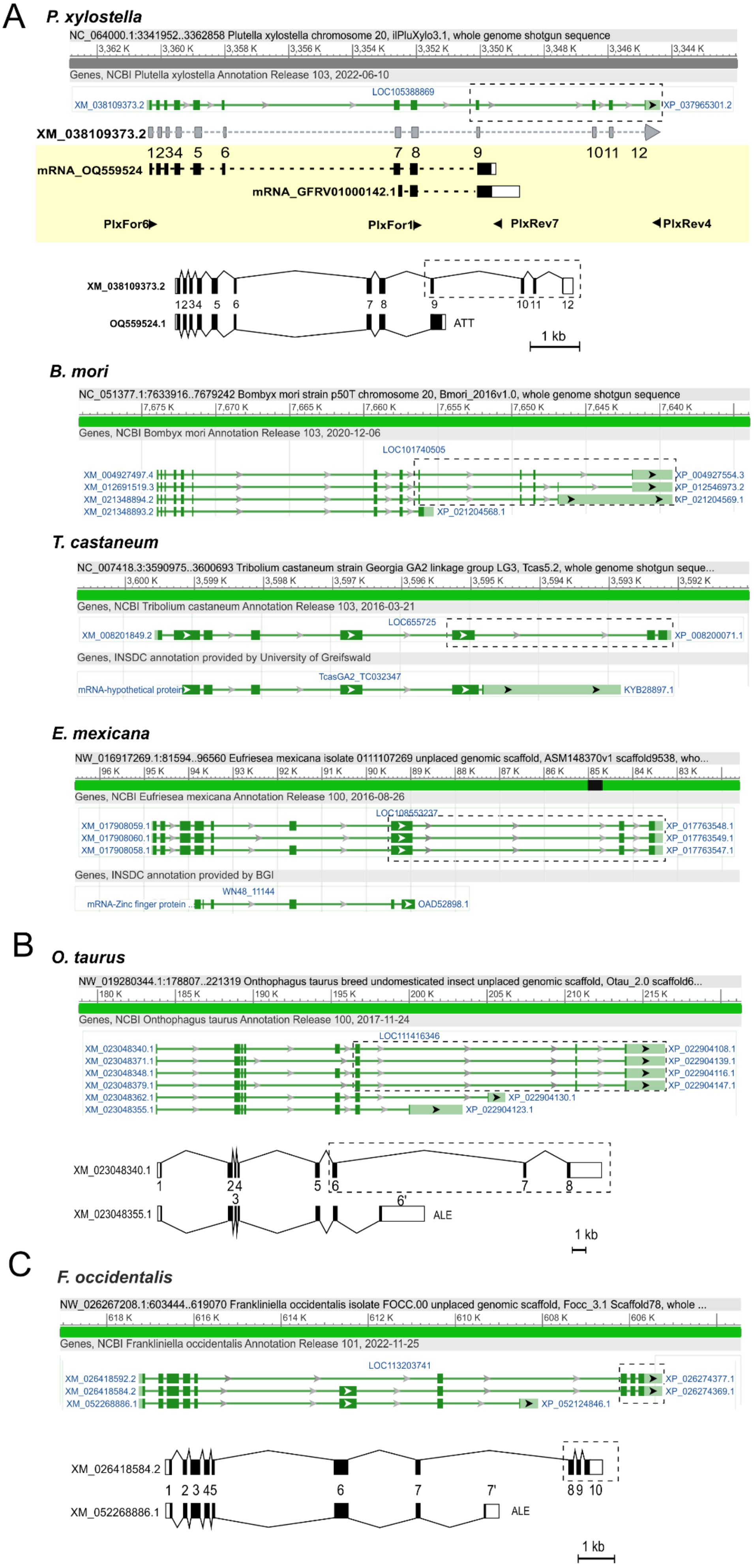

A
P. xylostella
NC_064000.1:3341952..3362858 Plutella xylostella chromosome 20, ilPluXylo3.1, whole genome shotgun sequence
Genes, NCBI Plutella xylostella Annotation Release 103, 2022-06-10
LOC105388869
XM_038109373.2
XP_037965301.2
XM_038109373.2
1234 5 6 7 8 9 1011 12
mRNA_OQ559524
mRNA_GFRV01000142.1
PlxFor6
PlxFor1
PlxRev7
PlxRev4
XM_038109373.2
1234 5 6 7 8 9 1011 12
OQ559524.1
ATT
1 kb
B. mori
NC_051377.1:7633916..7679242 Bombyx mori strain p50T chromosome 20, Bmori_2016v1.0, whole genome shotgun sequence
Genes, NCBI Bombyx mori Annotation Release 103, 2020-12-06
LOC101740505
XM_004927497.4
XM_012691519.3
XM_021348894.2
XM_021348893.2
XP_004927554.3
XP_012546973.2
XP_021204569.1
XP_021204568.1
T. castaneum
NC_007418.3:3590975..3600693 Tribolium castaneum strain Georgia GA2 linkage group LG3, Tcas5.2, whole genome shotgun seque...
Genes, NCBI Tribolium castaneum Annotation Release 103, 2016-03-21
LOC655725
XM_008201849.2
XP_008200071.1
Genes, INSDC annotation provided by University of Greifswald
TcasGA2_TC032347
mRNA-hypothetical protein
KYB28897.1
E. mexicana
NW_016917269.1:81594..96560 Eufriesea mexicana isolate 0111107269 unplaced genomic scaffold, ASM148370v1 scaffold9538, who...
Genes, NCBI Eufriesea mexicana Annotation Release 100, 2016-08-26
LOC108553237
XM_017908059.1
XM_017908060.1
XM_017908058.1
XP_017763548.1
XP_017763549.1
XP_017763547.1
Genes, INSDC annotation provided by BGI
WN48_11144
mRNA-Zinc finger protein ..
OAD52898.1
B
O. taurus
NW_019280344.1:178807..221319 Onthophagus taurus breed undomesticated insect unplaced genomic scaffold, Otau_2.0 scaffold6...
Genes, NCBI Onthophagus taurus Annotation Release 100, 2017-11-24
LOC111416346
XM_023048340.1
XM_023048371.1
XM_023048348.1
XM_023048379.1
XM_023048362.1
XM_023048355.1
XP_022904108.1
XP_022904139.1
XP_022904116.1
XP_022904147.1
XP_022904130.1
XP_022904123.1
XM_023048340.1
1 24 3 5 6 7 8
6'
XM_023048355.1
ALE
1 kb
C
F. occidentalis
NW_026267208.1:603444..619070 Frankliniella occidentalis isolate FOCC.00 unplaced genomic scaffold, Focc_3.1 Scaffold78, whole ...
Genes, NCBI Frankliniella occidentalis Annotation Release 101, 2022-11-25
LOC113203741
XM_026418592.2
XM_026418584.2
XM_052268886.1
XP_026274377.1
XP_026274369.1
XP_052124846.1
XM_026418584.2
1 2 3 45 6 7 7' 89 10
XM_052268886.1
ALE
1 kb

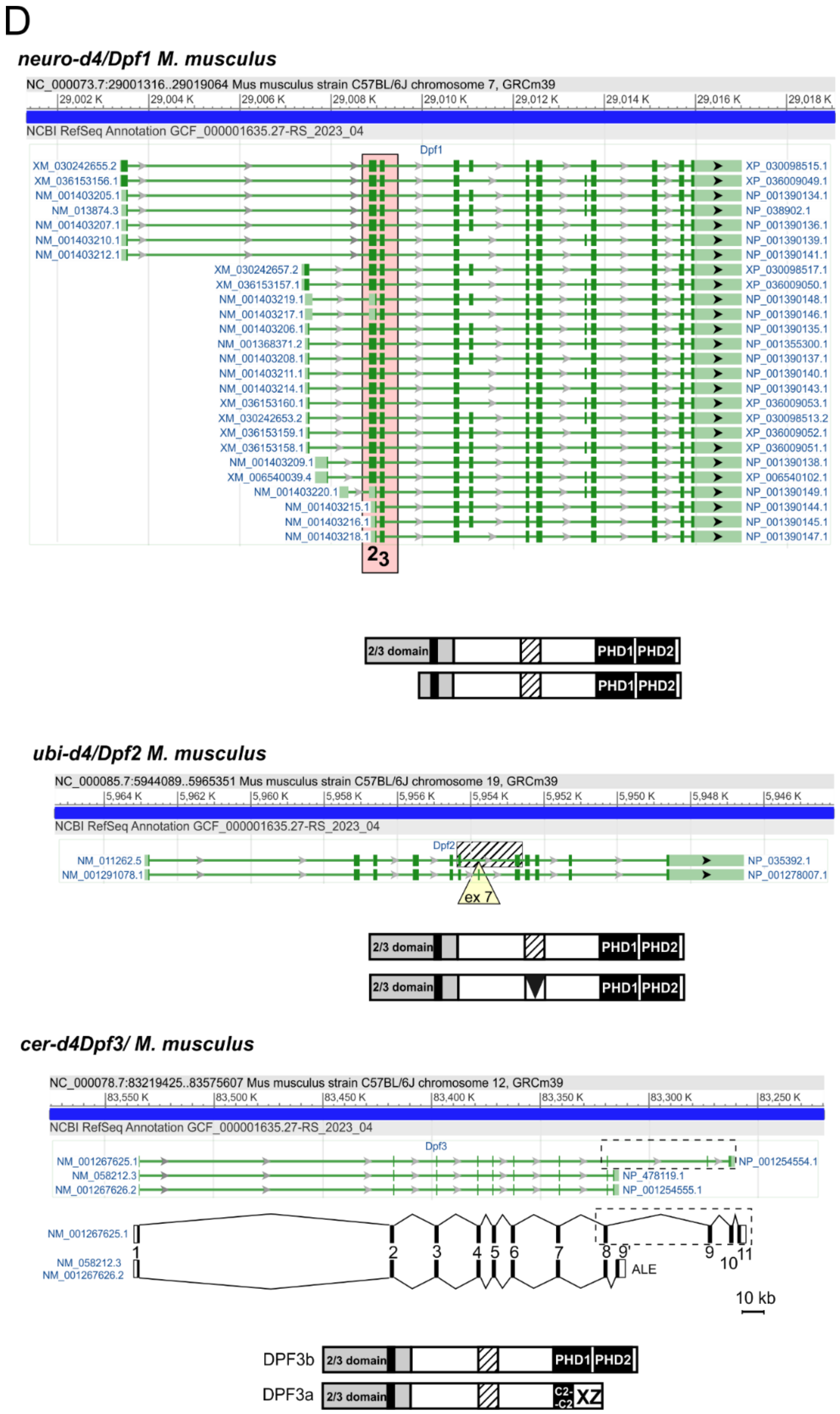

Figure S5. Splicing patterns of the *d4* genes in insects and mammals.

The maps of the *d4* loci (green plots) are shown according to the NCBI Genome Regions, Transcripts and Products Viewer. The maps of the *d4* family genes of *Onthophagus taurus* (LOC111416346) and *Frankliniella occidentalis* (LOC113203741) have been modified to show only the two alternative transcripts that are considered in this paper. The dashed boxes indicate exons encoding the D4 domain.

(A) The organization of d4 family gene *LOC105388869* in *Plutella xylostella*.

Currently, NCBI provides the single accession number XM_038109373.2 for the predicted transcript that encodes the full-length D4 protein.

The structures of the partial transcript from the NSBI TSA database (acc. # GFRV01000142.1) and the full ORF mRNA (GenBank acc. # OQ559524) obtained by RT-PCR in this study are shown in the yellow panel. The gray and black rectangles represent the exons 1 to 12. The PlxFor6, PlxRev4, PlxFor6, and PlxRev7 primers used for RT-PCR are shown by arrows. A schematic representation of the alternative splicing pattern of the gene is shown below. The isoform XM_038109373.2 includes exons 1-12. The alternatively spliced isoform OQ559524 exhibits exon 9 extension and alternative transcription termination (ATT). The isoform encodes DPF3a-like protein.
The similar splicing pattern to generate the DPF3a-like isoform is suggested by the maps of predicted transcripts of the *d4* genes in *Bombyx mori*, *Tribolium castaneum* and *Eufriesea mexicana* (shown below).
(B) The organization of *d4* family gene LOC111416346 in *Onthophagus taurus*. The two of the six predicted transcripts are shown on the locus map. A schematic representation of the alternative splicing pattern of the gene is shown below. The XM_023048340.1 isoform, which encodes the full-length D4 protein, includes exons 1-8. The alternative spliced isoform XM_023048355.1 includes exons 1-6 and alternative last exon 9 (ALE). The isoform encodes DPF3a-like protein.
(C) The organization of *d4* family gene LOC113203741 in *Frankliniella occidentalis*. The two of the three predicted transcripts are shown on the locus map. A schematic representation of the alternative splicing pattern of the gene is shown below. The XM_026418584.2 isoform, which encodes the full-length D4 protein, includes exons 1-10. The alternative spliced isoform XM_052268886.1 includes exons 1-7 and alternative last exon 11 (ALE). All exons encoding the D4 domain are excluded from the isoform.
(D) The organization of *d4* family genes (*neuro-d4/Dpf1, ubi-d4/Dpf2, cer-d4Dpf3*) in *Mus musculus*. Pink box indicates the location of the exons 2 and 3 encoding the 2/3 domain. Alternative transcription starting from the non-coding exons in the *neuro-d4/Dpf1* gene leads to N-terminal truncation of the 2/3 domain in the encoded isoforms because translation starts at the end of exon 2. The yellow triangle indicates the location of the exon in the *ubi-d4/Dpf2* that is included in the alternatively spliced isoform, disrupting the Kruppel-type ZF. The dashed box indicates the location of the exons encoding the D4 domain in the *cer-d4Dpf3* gene. Alternative last exon splicing (ALE) results in the D4 domain deficient isoform DPF3a/XZ. A schematic representation of the domain organization of the protein products of the genes is shown below the maps. The 2/3 domain caring NLS (black bar) is filled with gray, the Kruppel-type zinc finger is shaded, the PHD fingers and a C2C2 motif of PHD1 are filled with black.
