# Supplementary material for: The Drosophila toothrin Gene Related to the d4 Family Genes: An Evolutionary View on Origin and Function
Source: Int J Mol Sci. 2024 Dec 13;25(24):13394. doi: 10.3390/ijms252413394 (PMC11678306; doi:10.3390/ijms252413394)
Supplement: Supplementary file 1 [file ijms-25-13394-s001.zip › Figure S6.pdf]

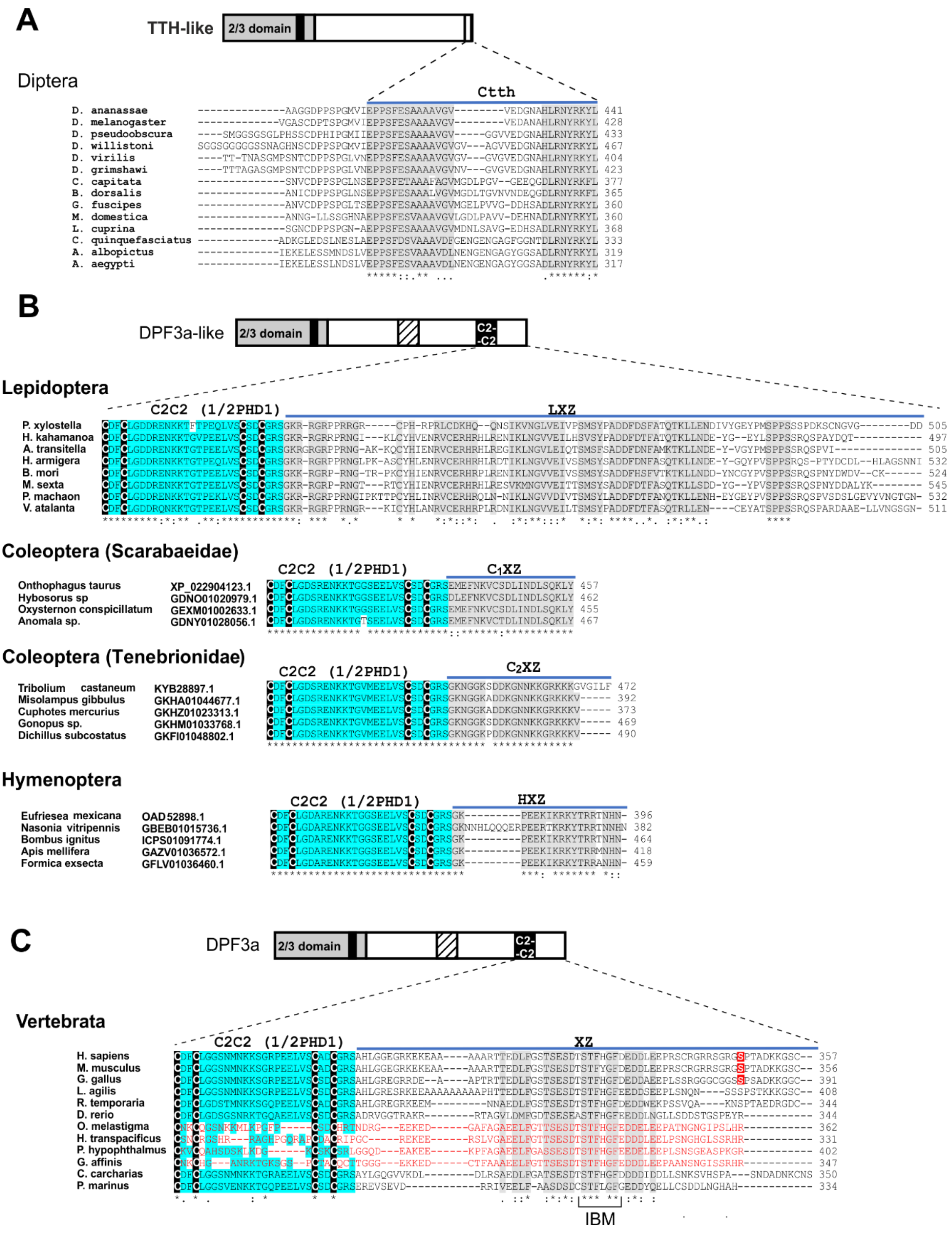

Figure S6. The specific C-tails of D4 related proteins lacking D4 domains.

The schematic images represent the domain organization TTH-like, DPF3a-like and DPF3a proteins.

The 2/3 domain carrying NLS (black bar) is filled with gray, the Kruppel-type zinc finger is shaded, the PHD fingers and a C2C2 motif of PHD1 (1/2PHD1) are filled with black. The alignment of C-terminal amino acid sequences is shown below. The taxon-specific C-tails are indicated by the blue line. The conserved amino acid residues of C2C2 motifs are highlighted in blue. Zinc-binding residues highlighted in black. The conserved residues of the taxon-specific C-tails are highlighted in gray. (*) – conserved residue, (:) – scoring > 0.5, (.) – scoring ≤ 0.5.

(A) The alignment of C-terminal amino acid sequences of the TTH-like proteins.

The species and the corresponding NCBI/GenBank accession numbers of the proteins are as follows: *Drosophila ananassae* XP_001966992.1, *D. melanogaster* NP_001285216.1, *D. pseudoobscura* XP_001354325.3, *D.*

willistoni XP_002071558.1, *D. virilis* XP_002055062.1, *D. grimshawi* XP_001995534.1, *Ceratitis capitata* XP_004529578.1, *Bactrocera dorsalis* XP_011202612.1, *Glossina fuscipes* XP_037887255.1, *Musca domestica* XP_005183185.1, *Lucilia cuprina* XP_023301960.1, *Culex quinquefasciatus* XP_038108115.1, *Aedes albopictus* XP_019526753.1, *Aedes aegypti* XP_001660913.1

(B) The alignment of C-terminal amino acid sequences of the insect DPF3a-like isoforms.

A list of species and the NCBI/GenBank accession numbers of the proteins and transcripts are shown on the right of the alignment charts.

Lepidoptera.

The Lepidoptera species and corresponding NCBI/GenBank protein accession numbers are as follows: *P. xylostella (Plutella xylostella,* WDY85960.1*), H. kahamanoa (Hyposmocoma kahamanoa* XP_026321161.1*), A. transitella (Amyelois transitella,* XP_013191758.1*), H. armigera (Helicoverpa armigera,* XP_049701608.1*), B. mori (Bombyx mori,* XP_021204568.1*), M. sexta (Manduca sexta,* XP_037298686.1*), P. machaon (Papilio Machaon,* XP_045540415.1*), V. atalanta (Vanessa atalanta,* XP_047540404.1*)*. The Lepidoptera-specific C-tails of the DPF3a-like isoforms are designated LXZ.

Coleoptera (Scarabaeidae)

The amino acid sequence of the predicted DPF3a-like isoform of ***Onthophagus taurus*** (NCBI/GenBank acc. #XP_022904123.1) was aligned with the deduced amino acid sequences from the Transcriptome Shotgun Assembly database (NCBI TSA) found in related **Scarabaeidae** species using the TBLASTN search. The transcripts contain full-length ORF of DPF3a-like isoform. The **Scarabaeidae**-specific C-tails of the DPF3a-like isoforms are designated C_1XZ.

Coleoptera (Tenebrionidae)

The amino acid sequence of the predicted DPF3a-like isoform of ***Tribolium castaneum*** (NCBI/GenBank acc. #KYB28897.1) was aligned with the deduced amino acid sequences from the Transcriptome Shotgun Assembly database (NCBI TSA) found using a TBLASTN search in the related **Tenebrionidae** species. The transcripts contain full ORF of DPF3a-like isoform. The **Tenebrionidae**-specific C-tails of the DPF3a-like isoforms are designated C_2XZ.

Hymenoptera

The amino acid sequence of the predicted DPF3a-like isoform of *Eufriesea mexicana* (NCBI/GenBank acc. # OAD52898.1) was aligned with the deduced amino acid sequences from the Transcriptome Shotgun Assembly database (NCBI TSA) found using the TBLASTN search in the related **Hymenoptera** species. The transcripts contain full ORF of DPF3a-like isoform. The **Hymenoptera**-specific C-tails of the DPF3a-like isoforms are designated HXZ.

(C) The alignment of C-terminal amino acid sequences of vertebrates DPF3a isoforms. The vertebrate-specific C-tails of the DPF3a isoforms are designated XZ. The amino acid sequences of the protein products of d4 family genes that encode in some fish only the DPF3a isoform are colored in red. The species and the corresponding NCBI/ GenBank accession numbers of the proteins are as follows: *H. sapiens* (*Homo sapiens,* NP_036206.3), *M. musculus* (*Mus musculus,* NP_478119.1), *G. gallus* (*Gallus Gallus,* XP_040556497.1*), L. agilis (Lacerta agilis,* XP_033002357.1*), R. temporaria (Rana temporaria* XP_040189606.1*), D. rerio (Danio rerio,* XP_005160801.1*), O. melastigma (Oryzias melastigma,* XP_024114574.1*), H. transpacificus* (*Hypomesus transpacificus,* XP_046905680.1), *P. hypophthalmus* (*Pangasianodon hypophthalmus,* XP_026789499.2), *G. affinis* (*Gambusia affinis,* XP_043999567.1), *C. carcharias (Carcharodon carcharias,* XP_041070480.1*), P. marinus (Petromyzon marinus,* XP_032803650.1*)*. The conserved IDB Binding Motif (IBM) and phosphorylation sites for Kasein kinase II (red shaded serines) are shown according to [14,15], respectively.
