# Supplementary material for: The Drosophila toothrin Gene Related to the d4 Family Genes: An Evolutionary View on Origin and Function
Source: Int J Mol Sci. 2024 Dec 13;25(24):13394. doi: 10.3390/ijms252413394 (PMC11678306; doi:10.3390/ijms252413394)
Supplement: Supplementary file 1 [file ijms-25-13394-s001.zip › Figure S7.pdf]

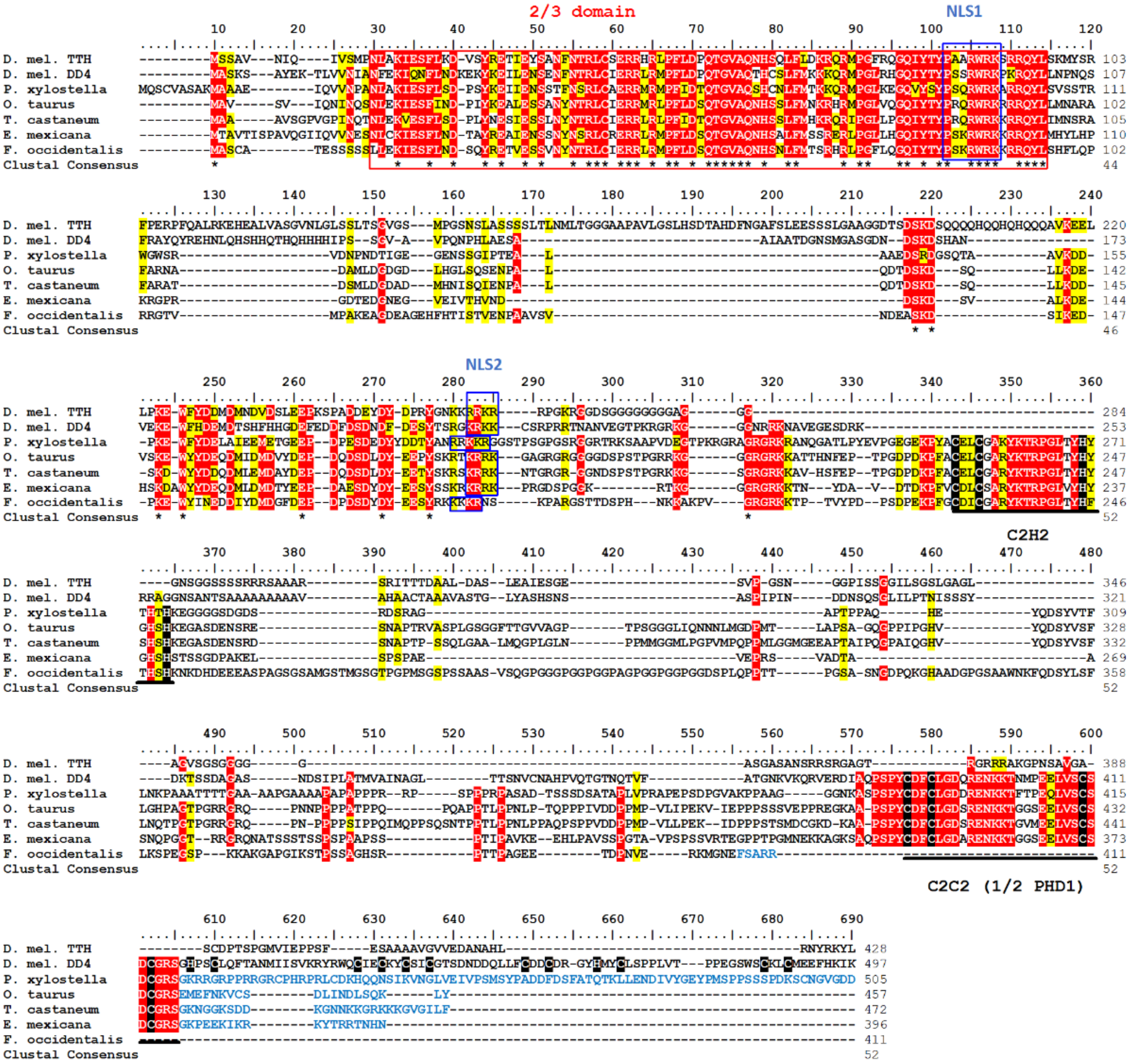

Figure S7. The similarity and divergence between *D. melanogaster* TTH and DD4 proteins and the DPF3a-like isoforms from the insect species of different orders. The alignment of *D. melanogaster* (Diptera) TTH and DD4 proteins, DPF3a-like isoforms of *P. xylostella* (Lepidoptera), *O. taurus* and *T. castaneum* (Coleoptera), *E. mexicana* (Hymenoptera) and isoform lacking D4 domain other than DPF3a of *F. occidentalis* (Thysanoptera). Species are as follows: *D. mel.* **(***Drosophila melanogaster*), *P. xyl***.** **(***Plutella xylostella*), *O. taurus* (*Onthophagus taurus*), *T. castaneum (Tribolium castaneum)***,** *E. mexicana* (*Eufriesea mexicana*), *F. occidentalis (Frankliniella occidentalis)*. The conserved amino acids are indicated by asterisks (*), identical residues are highlighted in red, the similar residues are highlighted in yellow (threshold for shading is 50%). The 2/3 domain is red-framed. Zinc-binding residues are highlighted in black. NLS1 and NLS2 are blue-framed. The C-tails of the DPF3a-like isoforms and other isoforms lacking the D4 domain are colored blue. C2H2 Kruppel –type ZF and C2C2 motif of the first PHD ZF (1/2 PHD1) of D4 domain are underlined.
