# Supplementary material for: The Drosophila toothrin Gene Related to the d4 Family Genes: An Evolutionary View on Origin and Function
Source: Int J Mol Sci. 2024 Dec 13;25(24):13394. doi: 10.3390/ijms252413394 (PMC11678306; doi:10.3390/ijms252413394)
Supplement: Supplementary file 1 [file ijms-25-13394-s001.zip › Figure S8.pdf]

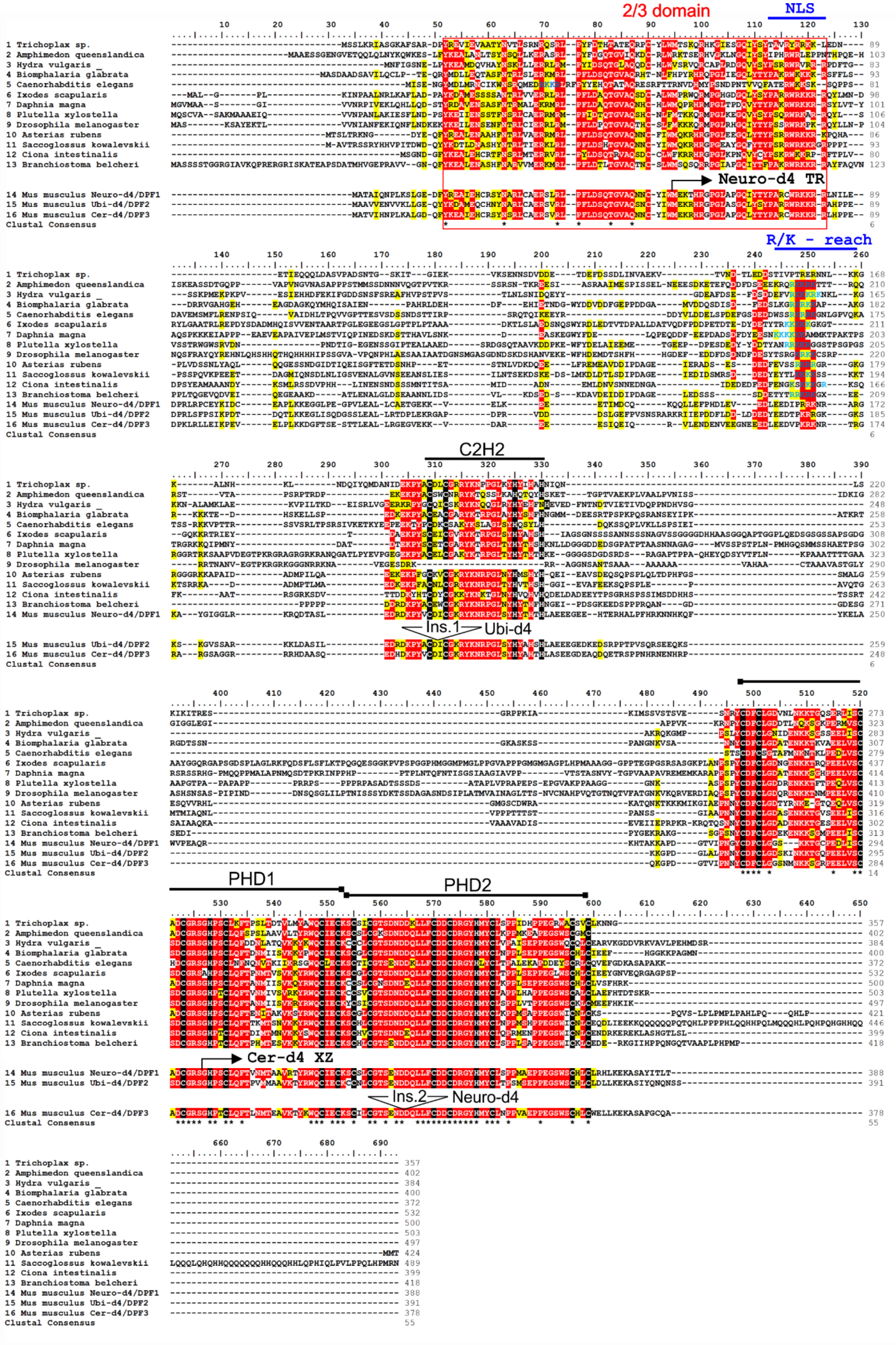
2/3 domain
NLS
Neuro-d4 TR
R/K - reach
C2H2
Ins.1
Ubi-d4
PHD1
PHD2
Cer-d4 XZ
Ins.2
Neuro-d4
1 Trichoplax sp.
2 Amphimedon queenslandica
3 Hydra vulgaris
4 Biomphalaria glabrata
5 Caenorhabditis elegans
6 Ixodes scapularis
7 Daphnia magna
8 Plutella xylostella
9 Drosophila melanogaster
10 Asterias rubens
11 Saccoglossus kowalevskii
12 Ciona intestinalis
13 Branchiostoma belcheri
14 Mus musculus Neuro-d4/DPF1
15 Mus musculus Ubi-d4/DPF2
16 Mus musculus Cer-d4/DPF3
Clustal Consensus

Neuro-d4 TR
>14_1_NP_001390149.1 zinc finger protein neuro-d4 isoform 16 (*Mus musculus*)
MEKTHRGPGLAPGQIYTYPARCWRKKRRLNILEDPRLRPCEYKIDCEAPLKKEGGLPEGPVLEALLCAETGEKKVEL
KEEETIMDCQKQQLLEFPHDLEVEDLEEDIPRRKNRARGKAYGIGGLRKRQDTASLEDRDKPYVCDICGKRYKNRP
GLSYHYTHTHLAEEEGEEHTERHALPFHRKNNHKQFYKELAWVPEAQRKHTAKKAPDGTVIPNGYCDFCLGGSK
KTGCPEDLISCADCGRSGHPSCLQFTVNMTAAVRTYRWQCIECKSCSLCGTSENDDQLLFCDDCDRGYHMYCLSPP
MAEPPEGSWSCHLCLRHLKEKASAYITLT
Ins 1 Ubi-d4
>15_1_NP_001278007.1 zinc finger protein ubi-d4 isoform 1 (*Mus musculus*)
MAAVVENVVKLLGEQYYKDAMEQCHNYNARLCAERSVRLPFLDSQTGVAQSNCYIWMEKRHRGPGLASGQLYS
YPARRWRKKRRAHPPEDPRLSFPSIKPDTDQTLKKEGLISQDGSSLEALLRTDPLEKRGAPDPRVDDDSLGEFPVSNS
RARKRIIEPDDFLDDLDDEDYEEDTPKRRGKGKSKSKGVSSARKKLDASILEDRDKPYACDNSFKQKHTSKAPQRVC
GKRYKNRPGLSYHYAHSHLAEEEGEDKEDSRPPTPVSQRSEEQKSKKGPDGLALPNNYCDFCLGDSKINKKTGQPE
ELVSCSDCGRSGHPSCLQFTPVMMAAVKTYRWQCIECKCCNLCGTSENDDQLLFCDDCDRGYHMYCLTPSMSEPP
EGSWSCHLCLDLLKEKASIYQNQNSS
Ins 2 Neuro-d4
>14_2_NP_001390134.1 zinc finger protein neuro-d4 isoform 3 (*Mus musculus*)
MATAIQNPLKSLGEDFYREAIEHCRSYNARLCAERSLRLPFLDSQTGVAQNNCYIWMEKTHRGPGLAPGQIYTYPA
RCWRKKRRLNILEDPRLRPCEYKIDCEAPLKKEGGLPEGPVLEALLCAETGEKKVELKEEETIMDCQKQQLLEFPHD
LEVEDLEEDIPRRKNRARGKAYGIGGLRKRQDTASLEDRDKPYVCDICGKRYKNRPGLSYHYTHTHLAEEEGEEHT
ERHALPFHRKNNHKQFYKELAWVPEAQRKHTAKKAPDGTVIPNGYCDFCLGGSKKTGCPEDLISCADCGRSGHPS
CLQFTVNMTAAVRTYRWQCIECKSCSLCGTSENDGASWAGLTPQDQLLFCDDCDRGYHMYCLSPPMAEPPEGSW
SCHLCLRHLKEKASAYITLT
XZ Cer-d4
>16_1_NP_001254555.1 zinc finger protein DPF3 isoform 2 (*Mus musculus*)
MATVIHNPLKALGDQFYKEAIEHCRSYNSRLCAERSVRLPFLDSQTGVAQNNCYIWMEKRHRGPGLAPGQLYTYP
ARCWRKKRRLHPPEDPKLRLLEIKPEVELPLKKDGFTSESTTLEALLRGEGVEKKVDAREEESIQEIQRVLENDENVE
EGNEEEDLEEDVPKRKNRTRGRARGSAGGRRRHDAASQEDHDKPYVCDICGKRYKNRPGLSYHYAHTHLASEEG
DEAQDQETRSPPNHRNENHRPQKGPDGTVIPNNYCDFCLGGSNMNKKSGRPEELVSCADCGRSAHLGGEGRKEK
EAAAAARTTEDLFGSTSESDTSTFYGFDEDDLEEPRSCRGRRSGRGSPTADKKGSC

Figure S8. Evolutionary conservation of the domains in D4 family proteins.

Multiple sequence alignment of protein products of *d4* family genes from different animal species. Identical a.a. residues are highlighted in red, similar a.a. residues are highlighted in yellow with 50% threshold for shading/coloring. Zinc coordinating amino acids are highlighted in black. Designations: 2/3 domain is in red frame; NLS – position of putative nuclear localisation signal; R/K reach-conserved stretch of positive charged a.a. (in some D4 family proteins contains additional NLS); C2H2 – position of Kruppel-type zink finger; PHD1,PHD2 – positions of PHD-type zink fingers, parts of D4 domain; Neuro-d4 TR – Start of the sequence of N-terminally truncated mouse *Neuro-d4* splice isoform; Ins-1 Ubi-d4 – a 14 a.a. insert into C2H2 Kruppel-type ZF generated by exon inclusion splicing in mouse Ubi-d4 splice isoform; Ins2 – a 10 a.a. insert into PHD2 ZF generated by exon inclusion splicing in mouse *Neuro-d4* splice isoform; Cer-d4 XZ – C-terminus in DPF3a/XZ splice isoform, generated by alternative last exon in mouse *Cer-d4*. Below the alignment, the sequences of the splice isoforms are shown, with insertions and truncations highlighted in yellow.

Species are as follows: 1) *Trichoplax sp.*, RDD40009.1; 2) *Amphimedon queenslandica*, XP_019851835.1; 3) *Hydra vulgaris*, XP_002160159.3; 4) *Biomphalaria glabrata*, XP_013094012; *Caenorhabditis elegans*, 5) NP_498281.2; *Ixodes scapularis*, 6) XP_029835063.2; 7) *Daphnia magna*, XP_032791427.2; 8) *Plutella xylostella*, XP_037965301.2; 9) *Drosophila melanogaster*,DD4, NP_610163.1; 10) *Asterias rubens*, XP_033626936.1; 11) *Saccoglossus kowalevski*i, XP_006825505.1; 12) *Ciona intestinalis*, XP_018669421; 13) *Branchiostoma belcheri*, XP_019624796.1; 14) *Mus musculus*, NP_038902.1; 15) *Mus musculus*, NP_035392.1; 16) *Mus musculus*, NP_001254554.
