# Supplementary material for: The Drosophila toothrin Gene Related to the d4 Family Genes: An Evolutionary View on Origin and Function
Source: Int J Mol Sci. 2024 Dec 13;25(24):13394. doi: 10.3390/ijms252413394 (PMC11678306; doi:10.3390/ijms252413394)
Supplement: Supplementary file 1 [file ijms-25-13394-s001.zip › Table S1.pdf]

Supplemental Table S1: The copy number of *d4* family genes in metazoan species, as deduced from their nuclear genomes available in NCBI.

№	Species	Phyllum/Class	Gene Copy Number	NCBI/GenBank Gen Symbol	Genome Assembly	Annotation release	Updated on
**Protozoa**							
1	*Dictyostelium purpureum*	Amoebozoa/Dictyostelia	0	*DICPUDRAFT_15851*	GCF_000190715.1	v1.0	24-Oct-2023
2	*Monosiga brevicollis*	Choanoflagellata	0	*MONBRDRAFT_15569*	GCF_000002865.3	v1.0	2-May-2020
**№**	**Species**	**Phyllum/Class**	**Gene Copy Number**	**NCBI/GenBank Gen Symbol**	**Genome Assembly**	**Annotation release**	**Updated on**
**Metazoa**							
1	*Trichoplax adhaerens*	Placozoa/Uniplacotomia	1	*RDD40009.1*	GCA_000150275.1	v1.0	17-Jun-2008
2	*Amphimedon queenslandica*	Porifera/Demospongiae	1	*LOC100640749*	GCF_000090795.1	102	7-Nov-2023
3	*Hydra vulgaris*	Cnidaria/ Hydrozoa	1	*LOC100200083*	GCF_022113875.1	103	7-Sep-2022
4	*Nematostella vectensis*	Cnidaria/ Hexacorallia	1	*LOC5513005*	GCF_932526225.1	101	18-Aug-2023
5	*Exaiptasia diaphana*	Cnidaria/ Hexacorallia	1	*LOC110235691*	GCF_001417965.1	RS_2023_11	22-Nov-2023
6	*Acropora digitifera*	Cnidaria/ Hexacorallia	1	*LOC107334047*	GCF_000222465.1	100	12-Mar-2017
7	*Stylophora pistillata*	Cnidaria/ Hexacorallia	1	*LOC111336723*	GCF_002571385.1	100	5-Feb-2022
8	*Pocillopora damicornis*	Cnidaria/ Hexacorallia	1	*LOC113665136*	GCF_003704095.1	100	18-Aug-2023
9	*Orbicella faveolata*	Cnidaria/ Hexacorallia	1	*LOC110046080*	GCF_002042975.1	100	4-Feb-2022
10	*Dendronephthya gigantea*	Cnidaria/Octocorallia	1	*LOC114522302*	GCF_004324835.1	100	18-Feb-2023
**Protostomia**							
11	*Lingula anatina*	Brachiopoda/Lingulata	1	*LOC106164092*	GCF_001039355.2	101	21-Dec-2023

12	*Crassostrea gigas*	Mollusca/ Bivalvia	1	*LOC105345927*	GCF_902806645.1	102	14-Dec-2023
13	*Pecten maximus*	Mollusca/ Bivalvia	1	*LOC117324305*	GCF_902652985.1	100	14-Dec-2023
14	*Mizuhopecten yessoensis*	Mollusca/ Bivalvia	1	*LOC110446003*	GCF_002113885.1	100	21-Dec-2023
15	*Octopus sinensis*	Mollusca/Cephalopoda	1	*LOC115216323*	GCF_006345805.1	101	28-Dec-2023
16	*Octopus bimaculoides*	Mollusca/Cephalopoda	1	*LOC106867266*	GCF_001194135.2	RS_2023_01	23-Nov-2023
17	*Pomacea canaliculata*	Mollusca/Gastropoda	1	*LOC112553122*	GCF_003073045.1	100	9-Nov-2023
18	*Biomphalaria glabrata*	Mollusca/Gastropoda	1	*LOC106077827*	GCF_947242115.1	RS_2023_05	3-Apr-2024
19	*Aplysia californica*	Mollusca/Gastropoda	1	*LOC101853942*	GCF_000002075.1	102	14-Dec-2023
20	*Caenorhabditis elegans*	Nematoda/Chromadorea	1	***dpff-1***	GCF_000002985.6		23-Nov-2023
21	*Limulus polyphemus*	Arthropoda/Merostomata	3	*LOC106461931*	GCF_000517525.1	101	21-Dec-2023
				LOC106478371			
				LOC106459238			
22	*Tetranychus urticae*	Arthropoda/Arachnida	4	*LOC107371702*	GCF_000239435.1	101	22-Oct-2023
				LOC107359508			17-Mar-2021
				LOC107366194			22-Oct-2023
				LOC107367175			22-Oct-2023
23	*Panonychus citri*	Arthropoda/Arachnida	3	*LOC128397756*	GCF_014898815.1	RS_2023_01	21-Jun-2023
				LOC128395291			23-Nov-2023
				LOC128395295			23-Nov-2023
24	*Ixodes scapularis*	Arthropoda/Arachnida	1	*LOC8032246*	GCF_016920785.2	103	26-Mar-2024
25	*Galendromus occidentalis*	Arthropoda/Arachnida	1	*LOC100908887*	GCF_000255335.1	102	7-Dec-2023
26	*Centruroides sculpturatus*	Arthropoda/Arachnida	2	*LOC111625251*	GCF_000671375.1	100	22-Oct-2023
				LOC111640972			
27	*Stegodyphus dumicola*	Arthropoda/Arachnida	1	*LOC118194384*	GCF_010614865.1	100	22-Oct-2023

28	*Parasteatoda tepidariorum*	Arthropoda/Arachnida		1	*LOC107452497*	GCF_000365465.3	102	7-Dec-2023
29	*Daphnia magna*	Arthropoda/Branchiopoda		1	*LOC116928451*	GCF_020631705.1	101	30-Nov-2023
30	*Hyalella azteca*	Arthropoda/Malacostraca		2	*LOC108677705*<br>*LOC108672214*	GCF_000764305.2	101	10-Mar-2023<br>30-Nov-2023
31	*Penaeus japonicus*	Arthropoda/Malacostraca		2	***LOC122250070***<br>*LOC122250696*	GCF_017312705.1	100	7-Dec-2023
32	*Homarus americanus*	Arthropoda/Malacostraca		2	*LOC121878412*<br>***LOC121855616****	GCF_018991925.1	100	7-Dec-2023
33	*Portunus trituberculatus*	Arthropoda/Malacostraca		2	*LOC123516819*<br>***LOC123508560***	GCF_017591435.1	100	30-Nov-2023
34	*Eurytemora affinis*	Arthropoda/Copepoda		2	*LOC111700562*<br>*LOC111695774*	GCF_000591075.1	RS_2023_10	17-Oct-2023
			**Order**					
35	*Zootermopsis nevadensis*	Arthropoda/Insecta	Blattodea	1	***LOC110835538***	GCF_000696155.1	100	21-Dec-2023
36	*Frankliniella occidentalis*	Arthropoda/Insecta	Thysanoptera	1	***LOC113203741***	GCF_000697945.3	101	23-Nov-2023
37	*Acyrthosiphon pisum*	Arthropoda/Insecta	Hemiptera	1	*LOC100161288*	GCF_005508785.1	103	7-Dec-2023
38	*Cimex lectularius*	Arthropoda/Insecta		1	*LOC106672808*	GCF_000648675.2	101	28-Mar-2024
39	*Halyomorpha halys*	Arthropoda/Insecta		1	*LOC106686576*	GCF_000696795.2	101	9-Nov-2023
40	*Nilaparvata lugens*	Arthropoda/Insecta		1	*LOC111063959*	GCF_014356525.1	101	7-Dec-2023
41	*Athalia rosae*	Arthropoda/Insecta	Hymenoptera	1	*LOC105688831*	GCF_917208135.1	103	30-Nov-2023
42	*Nasonia vitripennis*	Arthropoda/Insecta		1	*LOC100122315*	GCF_009193385.2	104	14-Dec-2023

43	*Trichogramma pretiosum*	Arthropoda/Insecta		1	*LOC106659927*	GCF_000599845.2	101	22-Oct-2023
44	*Harpegnathos saltator*	Arthropoda/Insecta		1	*LOC105189949*	GCF_003227715.1	102	21-Dec-2023
45	*Camponotus floridanus*	Arthropoda/Insecta		1	*LOC105256570*	GCF_003227725.1	102	21-Dec-2023
46	*Solenopsis invicta*	Arthropoda/Insecta		1	*LOC105195058*	GCF_016802725.1	104	7-Dec-2023
47	*Acromyrmex echinatior*	Arthropoda/Insecta		1	*LOC105146934*	GCF_000204515.1	100	22-Oct-2023
48	*Dufourea novaeangliae*	Arthropoda/Insecta		1	*LOC107186025*	GCF_001272555.1	100	22-Apr-2023
49	*Megachile rotundata*	Arthropoda/Insecta		1	*LOC100877921*	GCF_000220905.1	101	22-Oct-2023
50	*Habropoda laboriosa*	Arthropoda/Insecta		1	*LOC108579766*	GCF_001263275.1	100	9-Nov-2023
51	*Eufriesea mexicana*	Arthropoda/Insecta		1	***LOC108553237***	GCF_001483705.1	100	9-Nov-2023
52	*Apis mellifera*	Arthropoda/Insecta		1	*LOC411629*	GCF_003254395.2	104	9-Nov-2023
53	*Apis florea*	Arthropoda/Insecta		1	*LOC100870729*	GCF_000184785.3	102	14-Dec-2023
54	*Bombus terrestris*	Arthropoda/Insecta		1	*LOC100646981*	GCF_910591885.1	103	30-Nov-2023
55	*Onthophagus taurus*	Arthropoda/Insecta		1	***LOC111416346***	GCF_000648695.1	100	22-Oct-2023
56	*Tribolium castaneum*	Arthropoda/Insecta		1	***LOC655725***	GCF_000002335.3	103	2-Nov-2023
57	*Tribolium madens*	Arthropoda/Insecta	Coleoptera	1	*LOC123014613*	GCF_015345945.1	100	30-Nov-2023
58	*Dendroctonus ponderosae*	Arthropoda/Insecta		1	*LOC109537683*	GCF_020466585.1	101	30-Nov-2023
59	*Leptinotarsa decemlineata*	Arthropoda/Insecta		1	*LOC111515412*	GCF_000500325.1	100	22-Oct-2023
60	*Plutella xylostella*	Arthropoda/Insecta		1	***LOC105388869***	GCF_932276165.1	103	30-Nov-2023
61	*Bombyx mori*	Arthropoda/Insecta		1	***LOC101740505***	GCF_030269925.1	RS_2024_01	12-Feb-2024
62	*Manduca sexta*	Arthropoda/Insecta	Lepidoptera	1	***LOC115442980***	GCF_014839805.1	102	14-Dec-2023
63	*Hyposmocoma kahamanoa*	Arthropoda/Insecta		1	***LOC113231186***	GCF_003589595.1	100	23-Oct-2023
64	*Amyelois transitella*	Arthropoda/Insecta		1	***LOC106135903***	GCF_032362555.1	RS_2023_11	30-Nov-2023
65	*Helicoverpa armigera*	Arthropoda/Insecta		1	***LOC110382479***	GCF_030705265.1	RS_2024_03	29-Mar-2024

66	*Papilio machaon*	Arthropoda/Insecta		1	***LOC106714730***	GCF_912999745.1	101	30-Nov-2023
67	*Vanessa atalanta*	Arthropoda/Insecta		1	***LOC125073578***	GCF_905147765.1	100	30-Nov-2023
68	*Ctenocephalides felis*	Arthropoda/Insecta	Siphonaptera	1	*LOC113382843*	ASM342690v1 (GCF_003426905.1)	100	10-May-2024
69	*Ceratitis capitata*	Arthropoda/Insecta	Diptera	2	*LOC101448984*<br>*LOC101457923*	GCF_000347755.3	103	22-Oct-2023<br>22-Apr-2022
70	*Bactrocera dorsalis*	Arthropoda/Insecta		2	*LOC105229176*<br>*LOC105225711*	GCF_023373825.1	103	30-Nov-2023<br>18-Aug-2023
71	*Glossina fuscipes*	Arthropoda/Insecta		2	*LOC119642222*<br>*LOC119636142*	GCF_014805625.1	100	26-Mar-2024
72	*Musca domestica*	Arthropoda/Insecta		2	*LOC101890649*<br>*LOC101901527*	GCF_030504385.1	RS_2023_08	23-Oct-2023<br>22-Sep-2023
73	*Lucilia cuprina*	Arthropoda/Insecta		2	*LOC111687646*<br>*LOC111684072*	GCF_022045245.1	101	30-Nov-2023<br>16-Feb-2023
74	*Drosophila suzukii*	Arthropoda/Insecta		2	*LOC108007830*<br>*LOC108013735*	GCF_013340165.1	102	14-Dec-2023<br>4-Apr-2023
75	*Drosophila melanogaster*	Arthropoda/Insecta		2	*d4*<br>*tth*	GCF_000001215.4	6.54	29-Dec-2023<br>25-Jan-2024
76	*Drosophila eugracilis*	Arthropoda/Insecta		2	*LOC108104156*<br>*LOC108118381*	GCF_018153835.1	102	7-Dec-2023
77	*Drosophila elegans*	Arthropoda/Insecta		2	*LOC121466748*<br>*LOC108137702*	GCF_018152505.1	102	9-Oct-2021<br>21-May-2021
78	*Drosophila ficusphila*	Arthropoda/Insecta		2	*LOC108095528*	GCF_018152265.1	102	7-Dec-2023

					LOC108097652			4-Apr-2023
79	*Drosophila kikkawai*	Arthropoda/Insecta		2	*LOC108085924*<br>*LOC108078998*	GCF_018152535.1	102	7-Dec-2023<br>4-Apr-2023
80	*Drosophila ananassae*	Arthropoda/Insecta		2	*LOC6493888*<br>*LOC6504484*	GCF_017639315.1	102	30-Nov-2023<br>4-Apr-2023
81	*Drosophila pseudoobscura*	Arthropoda/Insecta		2	*LOC4803327*<br>*LOC4814216*	GCF_009870125.1	104	14-Dec-2023<br>4-Apr-2023
82	*Drosophila virilis*	Arthropoda/Insecta		2	*LOC6626273*<br>*LOC6631538*	GCF_003285735.1	103	14-Dec-2023<br>13-Sep-2023
83	*Drosophila grimshawi*	Arthropoda/Insecta		2	*LOC6559867*<br>*LOC6569418*	GCF_018153295.1	103	7-Dec-2023<br>12-Sep-2023
84	*Drosophila willistoni*	Arthropoda/Insecta		2	*LOC6637923*<br>*LOC6649088*	GCF_018902025.1	102	30-Nov-2023<br>4-Apr-2023
85	*Culex quinquefasciatus*	Arthropoda/Insecta		2	*LOC6048692*<br>*LOC6052655*	GCF_015732765.1	100	26-Mar-2024
86	*Aedes aegypti*	Arthropoda/Insecta		2	*LOC5574646*<br>*LOC5573515*	GCF_002204515.2	101	23-Mar-2024
87	*Aedes albopictus*	Arthropoda/Insecta		2	*LOC109417245*<br>*LOC109398797*	GCF_035046485.1	RS_2024_01	15-Feb-2024<br>9-Feb-2024
88	*Anopheles gambiae str.*	Arthropoda/Insecta		1	*LOC1274933*	GCF_943734735.2	RS_2023_12	31-Jan-2024
89	*Anopheles coluzzii*	Arthropoda/Insecta		1	*LOC120950477*	GCF_943734685.1	101	28-Mar-2024
90	*Anopheles arabiensis*	Arthropoda/Insecta		1	*LOC120894851*	GCF_016920715.1	100	26-Mar-2024
91	*Anopheles merus*	Arthropoda/Insecta		1	*LOC121593110*	GCF_017562075.2	100	27-Mar-2024

92	*Anopheles stephensi*	Arthropoda/Insecta		1	*LOC118511956*	GCF_013141755.1	100	27-Mar-2024
93	*Anopheles albimanus*	Arthropoda/Insecta		1	*LOC118463574*	GCF_013758885.1	100	26-Mar-2024
94	*Anopheles nili*	Arthropoda/Insecta		1	*LOC128727580*	GCF_943737925.1	RS_2023_02	28-Mar-2024
95	*Anopheles cruzii*	Arthropoda/Insecta		1	*LOC128268990*	GCF_943734635.1	RS_2023_02	28-Mar-2024
96	*Anopheles maculipalpis*	Arthropoda/Insecta		1	*LOC126562026*	GCF_943734695.1	100	28-Mar-2024
97	*Anopheles darlingi*	Arthropoda/Insecta		1	*LOC125958079*	GCF_943734745.1	100	27-Mar-2024
98	*Anopheles funestus*	Arthropoda/Insecta		1	*LOC125770662*	GCF_943734845.2	100	28-Mar-2024
99	*Anopheles marshallii*	Arthropoda/Insecta		1	*LOC128712109*	GCF_943734725.1	RS_2023_02	28-Mar-2024
100	*Anopheles aquasalis*	Arthropoda/Insecta		1	*LOC126577087*	GCF_943734665.1	100	27-Mar-2024
101	*Anopheles moucheti*	Arthropoda/Insecta		1	*LOC128305395*	GCF_943734755.1	RS_2023_02	28-Mar-2024
**Deuterostomia**								
102	*Asterias rubens*	Echinodermata/ Asteroidea		1	*LOC117289848*	GCF_902459465.1	100	14-Dec-2023
103	*Acanthaster planci*	Echinodermata/ Asteroidea		1	*LOC110979454*	GCF_001949145.1	100	9-Nov-2023
104	*Strongylocentrotus purpuratus*	Echinodermata/ Echinoidea		1	*LOC583658*	GCF_000002235.5	102	14-Dec-2023
105	*Saccoglossus kowalevskii*	Hemichordata/ Enteropneusta		1	*Cer-d4*	GCF_000003605.2	101	5-Feb-2022
106	*Branchiostoma floridae*	Chordata/Leptocardii		1	*LOC118409378*	GCF_000003815.2	100	14-Dec-2023
107	*Branchiostoma belcheri*	Chordata/Leptocardii		1	*LOC109470323*	GCF_001625305.1	100	21-Dec-2023
108	*Ciona intestinalis*	Chordata/Ascidiacea		1	*zf(c2h2)-17*	GCF_000224145.3	104	21-Dec-2023
109	*Petromyzon marinus*	Chordata/Hyperoartia		2	***LOC116939432***<br>*LOC116954017*	GCF_010993605.1	100	14-Dec-2023
110	*Carcharodon carcharias*	Chordata/Chondrichthyes		3	*dpf1*	GCF_017639515.1	100	22-Mar-2024

				LOC121274703			7-Dec-2023
				LOC121292505			7-Dec-2023
111	*Hemiscyllium ocellatum*	Chordata/Chondrichthyes	3	*LOC132836090*	GCF_020745735.1	RS_2023_11	30-Nov-2023
				dpf1			29-Mar-2024
				dpf3			29-Mar-2024
112	*Danio rerio*	Chordata/Actinopterygii	4	*dpf1*	GCF_000002035.6	106	27-Mar-2024
				dpf2			
				dpf2l			
				dpf3			
113	*Oryzias melastigma*	Chordata/Actinopterygii	4	*dpf1*	GCF_002922805.2	101	15-Mar-2024
				dpf2			
				dpf2l			
				dpf3			
114	*Hypomesus transpacificus*	Chordata/Actinopterygii	5	*dpf1*	GCF_021917145.1	100	22-Mar-2024
				dpf3			22-Mar-2024
				LOC124468308			30-Nov-2023
				LOC124484403			30-Nov-2023
				LOC124487401			30-Nov-2023
115	*Pangasianodon hypophthalmus*	Chordata/Actinopterygii	5	*dpf1*	GCF_027358585.1	RS_2023_02	15-Mar-2024
				dpf2			15-Mar-2024
				dpf2l			15-Mar-2024
				dpf3			15-Mar-2024
				LOC113538555			23-Nov-2023
116	*Gambusia affinis*	Chordata/Actinopterygii	5	*dpf1*	GCF_019740435.1	100	22-Mar-2024

				dpf2			22-Mar-2024
				dpf3			22-Mar-2024
				LOC122836948			30-Nov-2023
				LOC122846584			30-Nov-2023
117	*Lacerta agilis*	Chordata/Reptilia	3	*DPF1*	GCF_009819535.1	100	19-Mar-2024
				DPF2			
				DPF3			
118	*Rana temporaria*	Chordata/Amphibia	3	*dpf1*	GCF_905171775.1	100	21-Mar-2024
				dpf2			
				dpf3			
119	*Gallus gallus*	Chordata/Aves	3	*DPF1*	GCF_016699485.2	106	12-Mar-2022
				DPF2			5-Mar-2024
				DPF3			5-Mar-2024
120	*Mus musculus*	Chordata/Mammalia	3	*Dpf1*	GCF_000001635.27	RS_2024_02	5-Mar-2024
				Dpf2			
				Dpf3			
121	*Homo sapiens*	Chordata/Mammalia	3	*DPF1*	GCF_000001405.40<br>GCF_009914755.1	RS_2023_03	5-Mar-2024
				DPF2			
				DPF3			

Date accessed; 01/04/2024

dd4-like

tth-like

Gene accession numbers **in bold** indicate that among the predicted protein product isoforms of these *d*4-related genes, the isoforms lacking the D4 domain were found.

Red-highlighted gene accession numbers indicate that these *d*4-related genes do not contain the D4 domain coding sequence.
